# The potential of CircRNA1002 as a biomarker in hepatitis B virus-related hepatocellular carcinoma

**DOI:** 10.7717/peerj.13640

**Published:** 2022-06-28

**Authors:** Ying Li, Ronghua Li, Da Cheng, Xiaoyu Fu, Lei Fu, Shifang Peng

**Affiliations:** 1 Department of Infectious Diseases, Xiangya Hospital, Central South University, Changsha Hunan, China; 2 Department of Nuclear Medicine, Xiangya Hospital, Central South University, Changsha Hunan, China

**Keywords:** Circular RNA, Hepatocellular carcinoma, Hepatitis B virus, Serum biomarker, mRNA

## Abstract

**Background:**

Although hepatocellular carcinoma (HCC) is the most common type of primary liver cancer, there is a lack of effective diagnostic measures. Circular RNAs (circRNAs) can be used as biomarkers for monitoring the occurrence and development of HCC. However, a convenient and reliable serum circRNA biomarker is not currently available.

**Materials & Methods:**

CircRNA expression profiles were explored using high-throughput sequencing technology, and targeted circRNAs and mRNAs were validated by quantitative reverse transcription PCR (RT-qPCR). The biological functions of circRNAs were investigated using Gene Ontology (GO) enrichment analysis and Kyoto Encyclopedia of Genes and Genomes (KEGG) pathway enrichment analysis. Downstream miRNAs and mRNAs of dysregulated circRNAs were predicted using TargetScan, miRanda, and miRDB; then circRNA-miRNA-mRNA interaction networks were constructed based on sequencing data and the Cancer Genome Atlas (TCGA).

**Results:**

A total of 50,327 circRNAs were identified, with 1,187 circRNAs significantly differentially expressed between hepatitis B virus (HBV)-related HCC and HBV asymptomatic carriers. Among these circRNAs, four (circRNA1002, circRNA7941, circRNA 39338, and circRNA44142) were validated by RT-qPCR as being statistically different either in HCC tissue or serum samples. circRNA1002 was significantly down-regulated in both HCC serum and tissue, indicating its reliability. Bioinformatics analysis showed that circRNA1002-associated genes were enriched in GO terms relating to hormone pathway and cell-cell interaction processes, which are involved in the progression of HCC.

**Conclusion:**

Our circRNA analysis of HCC patients and HBV asymptomatic carriers showed that circRNA1002 may be a reliable serum biomarker for HCC. These results could provide an improved assay for the early detection of HCC.

## Introduction

Globally, hepatocellular carcinoma (HCC) is the most common type of primary liver cancer and hepatitis B virus (HBV) is its primary etiological agent (Levrero & Zucman-Rossi, 2016; Yang et al., 2019). Surgical therapies such as liver hepatectomy and transplantation can significantly improve the prognosis of HCC patients, as well as their 5-year survival rate to 60–70% (Kotewall & Cheung, 2018; Kumari et al., 2018). Unfortunately, most patients cannot undergo therapeutic operations due to late diagnoses (Sun et al., 2020); therefore, detecting HCC in the early stage is crucial. There are many measures currently used for the early diagnosis of HCC. Liver biopsy is the gold standard, but it is not always the best choice since it is an invasive procedure. α-fetoprotein (AFP) is the most common biomarker used for HCC surveillance and diagnosis; however, it is not effective for the diagnosis of early liver cancer. In about 50% of early-stage HCC patients, serum AFP did not increase significantly (the cut off value was 20 ng/mL). Other diseases, such as liver cirrhosis, may cause an increase in AFP (Reichl & Mikulits, 2016; Zhang et al., 2014). Serum AFP, in combination with imaging detection methods such as liver ultrasonography or computed tomography (CT), can be used in early HCC detection. However, its diagnostic accuracy is poor, with sensitivity ranging from 41% to 65% and specificity ranging from 80% to 94% (Bruix, Reig & Sherman, 2016). Protein Induced by Vitamin K Absence or Antagonist-II (PIVKA-II) is another common biomarker used for early HCC detection and patients often have an increase in PIVKA-II when their AFP is negative (Luo et al., 2020). However, its expression is easily affected by hemolysis and jaundice (Kanazumi et al., 2000).

Liquid biopsy is also used for diagnostic purposes. In contrast to tissue biopsy, liquid biopsy collects body fluids such as blood (Rubis, Krishnan & Bebawy, 2019), and its key components include circulating tumor cells (CTCs) and circulating tumor DNA (ctDNA) (Ye et al., 2019). Exosomes are also promising diagnostic indicators used in liquid biopsy (Melo et al., 2015). Cancer research has sought to verify the diagnostic and monitoring value of these liquid biopsy indicators (Underwood et al., 2020), especially in HCC (Qi et al., 2018; Chen et al., 2020b). Liquid biopsy is non-invasive and can detect tumor dynamics (Wang et al., 2017a). However, it does have certain limitations. First, indicators have a short half-life; the average half-life of ctDNA is about 2 h (Diaz & Bardelli, 2014) and the average half-life of CTCs ranges from 1–2.4 h (Meng et al., 2004). Second, the small number of CTCs in serum and current detection technologies limit its clinical applications. It is also unclear if ctDNA reflects the representative characteristics of cancers (Wang et al., 2017a). Further studies are needed to find more effective and less invasive biomarkers.

Non-coding RNA is a potential biomarker of HCC, and circular RNAs (circRNAs) are a research focus (Wen, Zhou & Gu, 2020). CircRNAs are endogenous noncoding RNA that were first discovered in plant viroids (Sanger et al., 1976). Some circRNAs function by sponging to downstream microRNAs (miRNAs), although the function of most CircRNAs is unclear. Compared to linear non-coding RNA, CircRNAs have more advantages. They are more stable, have a longer half-life (>48 h) (Jeck & Sharpless, 2014; Enuka et al., 2016), and are abundantly expressed in many tissues (Wen, Zhou & Gu, 2020). CircRNAs have been studied in many cancers (Chen et al., 2017; He et al., 2017; Hsiao et al., 2017; Huang et al., 2019; Qiu et al., 2019a), such as liver cancer (Sun et al., 2020; Liu et al., 2019; Matboli et al., 2018; Wang et al., 2019a). CircRNAs have been shown to play a diagnostic and prognostic role in HCC (Sun et al., 2020). For example, Circ_104075 was suggested to be a promising diagnostic biomarker of HCC as it promoted HCC tumorigenesis and development by sponging miR-582 (Zhang et al., 2018b). Plasma hsa_circ_0001445 is a diagnostic and monitoring biomarker that affects HCC progression by inhibiting the proliferation, migration, and invasion of HCC cells (Zhang et al., 2018a). Han et al. (2017) investigated circRNA profiles in HCC tissues and found that circMTO1 was significantly downregulated in HCC tissues. HCC patients with low circMTO1 expression had reduced survival rates (Han et al., 2017). Given their high stability and abundant expression, circRNAs have the potential to be used as an HCC biomarker. However, they also have some possible limitations. Most studies have only focused on HCC cells or tissue samples, and it has not been determined whether these results can translate to clinical practice (Sartorius et al., 2019; Wang et al., 2019b). CircRNAs may express differently in blood and tissues, and reported circRNAs have only been verified in either serum or tissue samples; therefore, they lack reliability. Further studies on circRNA profiles are required, and a more promising and reliable circRNA biomarker must be found.

In this study, we analyzed the circRNA expression profiles in HCC, matched HBV asymptomatic carriers, and verified these carriers using RT-qPCR. Potential biological functions were determined and used to increase our knowledge of the epigenetic mechanisms of HCC.

## Methods and Materials

### Patients and samples

This study involved a total of 30 patients recruited between December 2019 and January 2020. In order to detect the circRNA profiles, we randomly selected nine whole blood samples: five samples from HBV-related HCC patients and four samples from HBV asymptomatic carriers. For the circRNA verification experiment, 10 blood samples were collected: five from HCC patients and five from HBV-asymptomatic carriers. We also collected paired tissue samples from 11 HBV-related HCC patients containing cancer and adjacent tissue for circRNA and mRNA verification. HCC samples were obtained from the Liver Surgery Department of Xiangya Hospital, Central South University, China, while HBV samples were collected from the outpatient department of Xiangya Hospital, Central South University. Detailed information is presented in Tables 1 and S1. Fresh tissue and blood samples were immediately stored at −80 °C after collection. The inclusion criteria for HCC cases in this study were as follows: (1) HCC without distant metastasis was diagnosed by a professional pathologist; (2) severe diseases such as heart and lung failure were excluded; and (3) no radiation therapy, chemotherapy, or other treatments were administered to these patients. All of the human HCC liver tissue and blood samples were collected under protocols approved by the Ethics Committee of Xiangya Hospital, Central South University (registration number: 201912537). The participants provided informed written consent prior to sample collection.

**Table 1:** Clinicopathological characteristics of 30 HCC patients and HBV asymptomatic carriers.

Characteristics	HCC (*n* = 21)	HBV (*n* = 9)
Gender		
Male	10	6
Female	11	3
Age (y)		
<50	12	8
≥50	9	1
AFP (ng/mL)		
<400	14	9
≥400	7	0
HBV DNA (IU/mL)		
≤10	2	1
>10	19	8
AST* (U/L)		
≤40	9	5
>40	12	4
ALT* (U/L)		
≤40	11	5
>40	10	4
Tumor size (cm)		
≤5	18	–
>5	3	–
Tumor number		
≤1	10	–
>1	11	–
TNM stage*		
I	10	–
II	8	–
III	3	–

**Note:**

* AST, Aspartate aminotransferase; ALT, Alanine aminotransferase; TNM, Tumor, Node, Metastasis.

### RNA library construction and sequencing

We collected whole blood (5 mL) samples from five HBV-related HCC patients and four HBV asymptomatic carriers. The samples were collected in PAXgene Blood RNA tubes (Qiagen, Hilden, Germany) and stored at −80 °C until they were used for RNA preparation. Total RNA was extracted using Trizol reagent (Invitrogen, Carlsbad, CA, USA) according to the manufacturer’s protocol. Approximately 5 ug of total RNA was used to deplete ribosomal RNA according to the manufacturer’s instructions for the Ribo-Zero™ rRNA Removal Kit (Illumina, San Diego, CA, USA). After removing ribosomal RNAs, the remaining RNAs were fragmented into small pieces using divalent cations under high temperature. The cleaved RNA fragments were then reverse-transcribed to create the cDNA, which were used to synthesize U-labeled second-stranded DNAs with *E. coli* DNA polymerase I, RNase H, and dUTP. An A-base was then added to the blunt ends of each strand to prepare them for ligation to the indexed adapters. Each adapter contained a T-base overhang for ligating the adapter to the A-tailed fragmented DNA. Single or dual-index adapters were ligated to the fragments, and size selection was performed with AMPureXP beads. After the heat-labile UDG enzyme treatment of the U-labeled second-stranded DNAs, the ligated products were amplified with PCR under the following conditions: initial denaturation at 95 °C for 3 min; eight cycles of denaturation at 98 °C for 15 s, annealing at 60 °C for 15 s, and extension at 72 °C for 30 s; and then final extension at 72 °C for 5 min. The average insert size for the final cDNA library was 300 bp (±50 bp). Finally, we performed the paired-end sequencing on an Illumina Hiseq 4000 (LC Bio,Hangzhou, China) following the manufacturer’s instructions.

### Bioinformatic analysis

The quality of the raw FASTQ reads was assessed using FastQC, and they were trimmed with trimmomatic (Bolger, Lohse & Usadel, 2014) for clean reads. These were aligned to a human reference genome (GRCh37/hg19) with BWA (Li, 2013). CircRNAs were identified using CIRCExplorer (Dong et al., 2019). Read counts for each circRNA were evaluated with htseq-count and the quantification of expression level was performed with Fragments Per Kilobase Million Reads (FPKM) (Anders, Pyl & Huber, 2015). The differential expression of circRNAs between the two groups was performed using the DESeq2 R package (Love, Huber & Anders, 2014). We defined differentially expressed circRNAs as those with a *P* value of < 0.05 and a |fold change| of ≥ 1.8. The RNA-seq power was 0.998, as calculated by RNASeqPower R package (version 1.34.0) with a sample size of four HBV asymptomatic carriers and five HBV-related HCC patients. To determine the potential functions of dysregulated circRNAs, we identified miRNAs that were able to bind to circRNAs based on their miRNA binding sites using miRanda (Enright et al., 2003) and TargetScan (http://targetscan.org). TargetScan and miRDB (http://mirdb.org/) were used to identify MiRNA target mRNAs and determine the potential biological function of circRNAs. Gene ontology (GO) enrichment analysis and Kyoto Encyclopedia of Genes and Genomes (KEGG) pathway enrichment analysis were performed with Clusterprofiler (Yu et al., 2012). Cytoscape (3.18.0) was used to visualize the final circRNA-miRNA-mRNA regulatory network (Saito et al., 2012).

### RT-qPCR validation of targeted circRNAs and mRNAs

RT-qPCR was performed to verify the predicted circRNAs and mRNAs. Briefly, total RNAs (1 ug) were transcribed into cDNAs using the SuperScript III Reverse Transcriptase kit (Invitrogen, Carlsbad, CA, USA). RT-qPCR was performed using Geneseed® qPCR SYBR® Green Master Mix and monitored using the ABI PRISM 7500 Sequence Detection System (Applied Biosystems, Waltham, MA, USA; Life Technologies, Carlsbad, CA, USA). Primers were synthesized by Qiagen (Valencia, CA, USA). GAPDH was used as an endogenous control gene. The RT-qPCR reaction conditions were as follows: hot start at 95 °C for 5 min, 40 cycles at 95 °C for 10 s, 60 °C for 34 s, melting curve stage at 95 °C for 15 s, 60 °C for 60 s, and 95 °C for 15 s. All of the reactions were carried out in triplicate. The experimental data were analyzed using the 2^−ΔΔCt^ method. The RT-qPCR primer sequences and circRNA sanger sequencing results are provided in the Supplemental Files.

### Statistical analysis

GraphPad Prism (version 7.0; GraphPad Software, La Jolla, CA, USA) was used for data analysis. Paired or unpaired *t*-tests were used to evaluate differences in expression levels between the two groups. A *P* value of < 0.05 was considered statistically significant.

## Results

### Identification of circRNA expression profiles in HCC patient and HBV asymptomatic carrier blood samples

**Figure 1:**
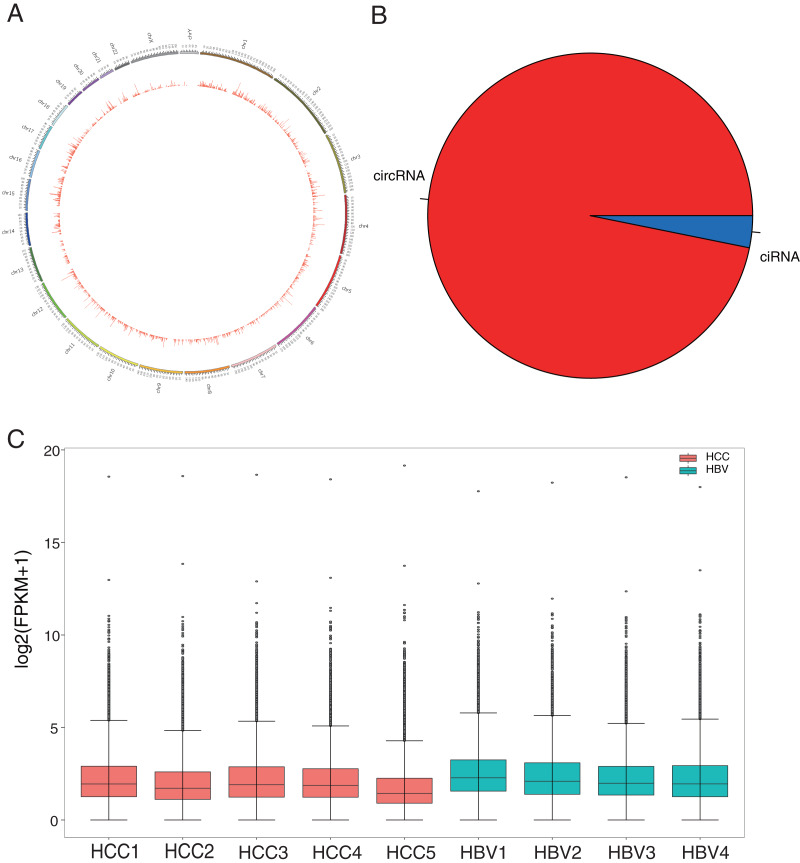
Identification and classification of circRNAs in HCC patients and HBV asymptomatic carriers. (A) Distribution of the 50,327 candidate circRNAs on the 24 chromosomes. (B) Percentage of intronic region-derived circRNA (ciRNA) and exonic region-derived circRNA (circRNA). (C) Expression level of candidate circRNAs in all nine samples.

HBV infection is a major risk factor for liver carcinogenesis (Kulik & El-Serag, 2019). To determine biomarkers in HBV-related HCC patients, RNA was collected from five HBV-related HCC patients and four HBV asymptomatic carriers. CircRNA profiles in liver tissues were examined based on nonribosomal RNA samples with RNA-seq. After mapping those clean reads to the reference genome, a total of 50,327 circRNAs were identified (Fig. 1A, Table S2). Based on their locations in the genome, 96.83% of the circRNAs were derived from exonic regions and the remaining 3.17% were derived from intronic regions (Fig. 1B). The expression levels of circRNAs were evaluated with FPKM and a median expression level from 1.42 to 3.18 (Fig. 1C). GO enrichment analysis showed that 7,240 circRNAs’ host genes were enriched in “covalent chromatin modification” and “histone modification” processes and located in “chromosomal region.” KEGG analysis revealed most host genes were enriched in “endocytosis” and “Shigellosis” (Fig. S1, Tables S3 and S4).

**Figure 2:**
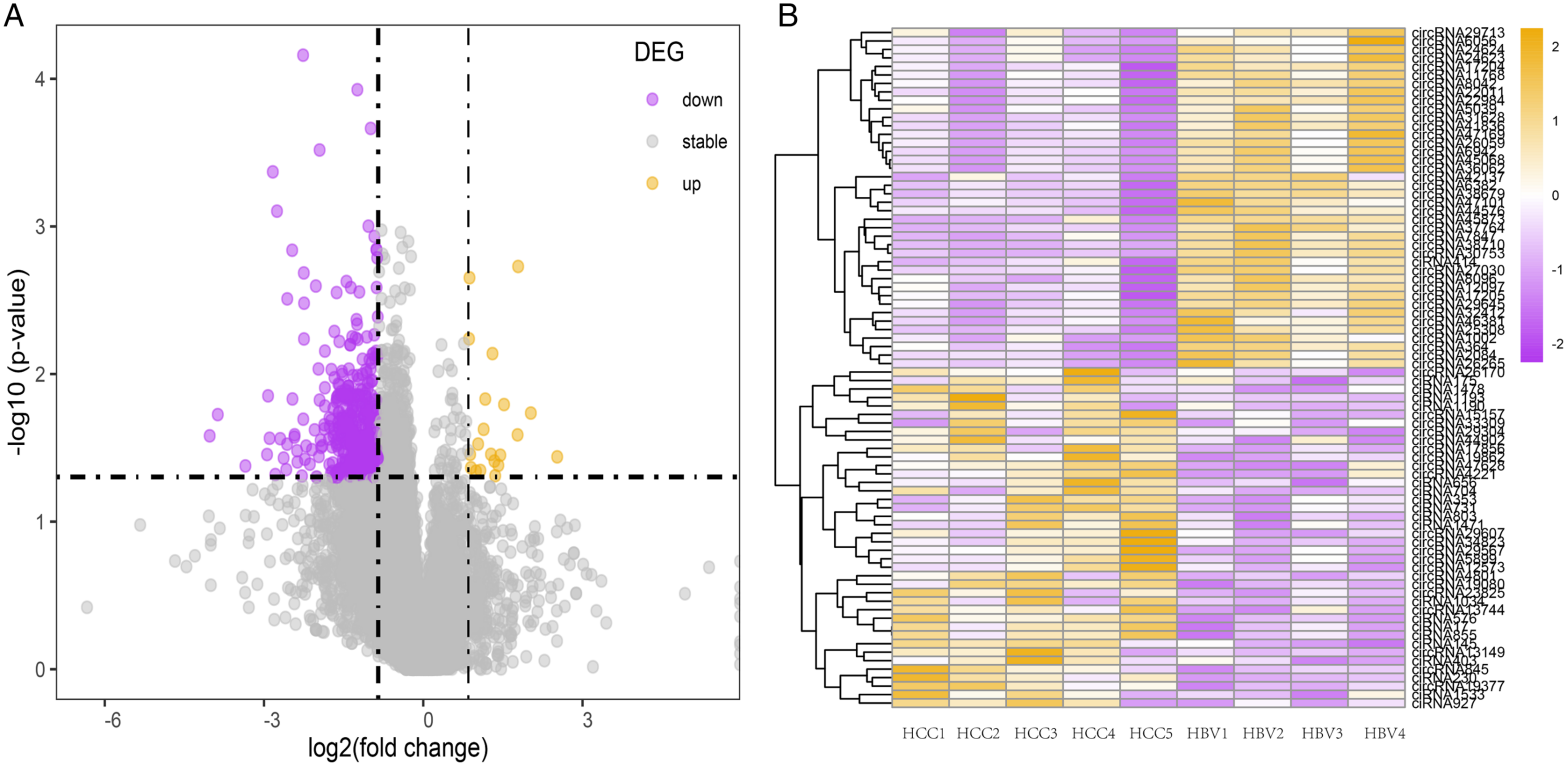
Summary of the differentially expressed circRNAs between HCC patients and HBV asymptomatic carriers. (A) Volcano plot showing the pattern of differentially expressed circRNAs. The purple and yellow dots represent the statistically significant up- and down-expressed circRNAs, respectively. The vertical lines indicate 1.8-fold upregulation and downregulation. The horizontal line represents a *P* value of 0.05. (B) Clustering heatmap of top 40 highly expressed DE-circRNAs in all nine samples. (HBV: hepatitis B asymptomatic carriers).

To investigate the potential role of circRNAs in HBV-related HCC, differentially expressed circRNAs were identified using DESeq2. Compared to the HBV asymptomatic carriers, a total of 1,131 circRNAs were significantly down-regulated in HBV-related HCC, with only 56 circRNAs significantly up-regulated (Figs. 2A and 2B, Table S5). The top 40 highly expressed circRNA differing between the HCC and HBV samples were shown *via* hierarchical clustering (Fig. 2B).

### Validation of target circRNAs detected in HCC and HBV asymptomatic carriers

**Figure 3:**
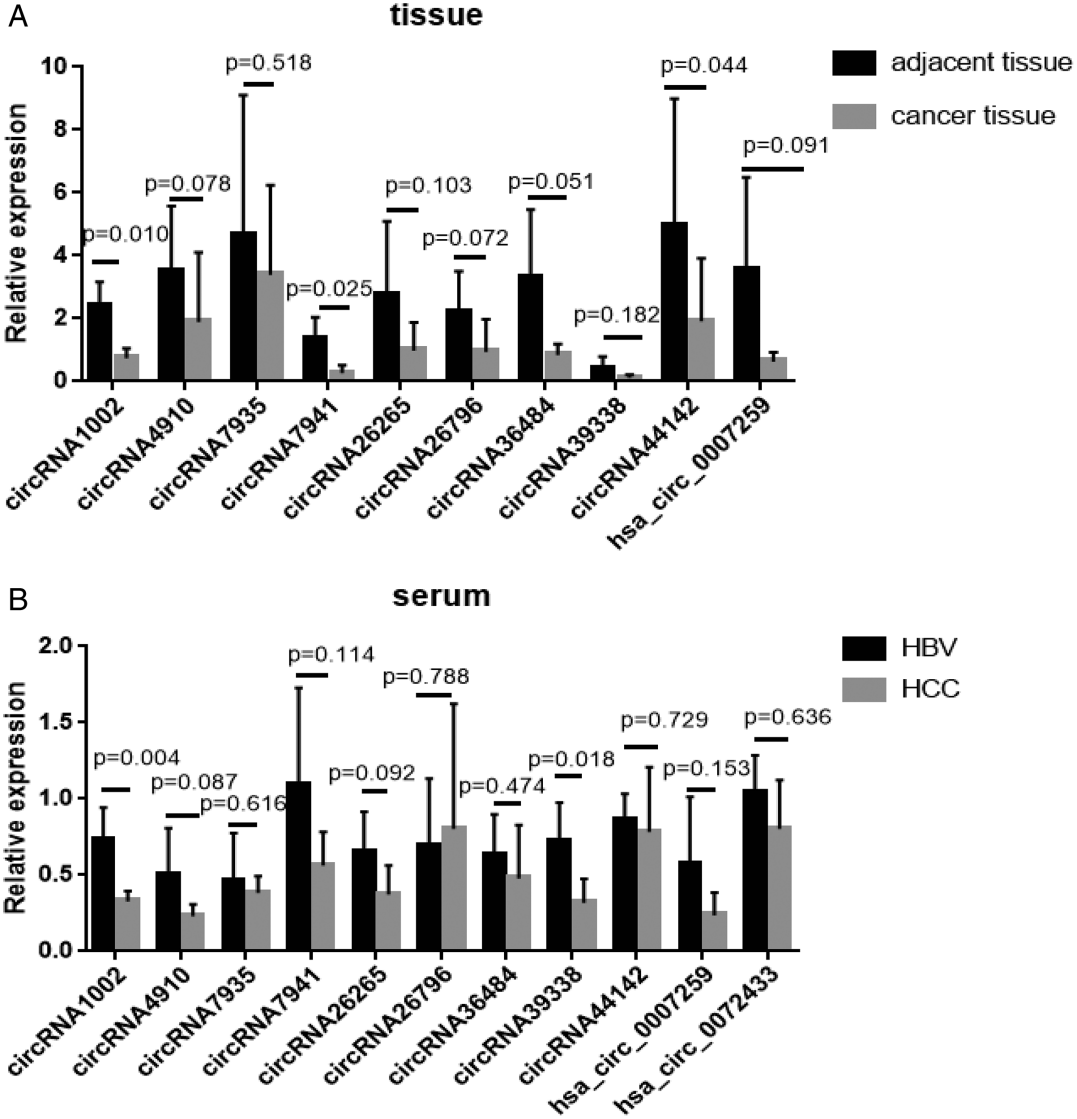
RT-qPCR validation of 11 selected circular RNAs. RT-qPCR validation of 11 selected circular RNAs. (A) Cancer *vs* adjacent non-disease tissue samples from HCC patients (*n* = 5, respectively). (B) Blood samples of HCC patients *vs* hepatitis B virus asymptomatic carriers (*n* = 5, respectively). The expression level of hsa_circ_0072433 in the tissue was too low to be calculated, so it is not shown.

Of all the detected circRNAs, 11 circRNAs were selected and verified by Sanger sequencing. The results are shown in Table S6. RT-qPCR was used to examine gene expression in HCC and matched HBV asymptomatic carriers (primers are shown in Table S7). These 11 circRNAs were selected based on previous findings. The detailed information is shown in Table 2. Among these, circRNA1002 (*P* = 0.010), circRNA7941 (*P* = 0.025), and circRNA44142 (*P* = 0.044) were significantly down-regulated in HCC samples compared to matched adjacent tissues (Fig. 3A).

**Table 2:** Basic characteristics of the 11 validated circRNAs.

CircRNA ID	Host gene name	Mean fpkm HCC*	Mean fpkm HBV^#^	*P* value	chr	Start	End
circRNA7941	MBOAT2	133.18	74.41	0.43	chr2	8,943,187	8,958,642
circRNA26265	CDC73	42.02	80.29	0.09	chr1	193,141,850	193,212,477
circRNA26796	KDM6A	82.53	178.56	0.14	chrX	44,961,284	45,020,730
circRNA4910	NRIP1	75.74	140.72	0.15	chr21	15,014,344	15,043,574
circRNA39338	AKT3	59.49	105.67	0.14	chr1	243,695,591	243,843,282
circRNA364	GTF2F2	54.69	104.65	0.07	chr13	45,151,687	45,207,505
circRNA44142	PPP2R5C	44.84	85.88	0.11	chr14	101,856,686	101,912,473
circRNA36484	BRE	57.76	109.68	0.15	chr2	27,987,993	28,129,380
circRNA1002	MED13L	42.30	76.98	0.07	chr12	116,096,669	116,111,512
circRNA7935	LINC00299	40.98	69.24	0.17	chr2	8,243,565	8,287,558
circRNA35761	PARP8	50.10	90.11	0.14	chr5	50,759,643	50,797,233

**Notes:**

* Expression level in HBV-related HCC patients.

# Expression level in HBV asymptomatic carriers.

The expression of these circRNAs was investigated in blood samples from HCC patients and HBV asymptomatic carriers. CircRNA1002 (*P* = 0.004) and circRNA39338 (*P* = 0.018) were also significantly down-regulated in the blood samples from HCC patients, which was consistent with our RNA sequencing results (Fig. 3B). Combined with the expression levels from the tissue and blood samples, circRNA1002 was selected as a potential biomarker for HCC.

### CircRNA-miRNA-mRNA interaction network revealed the biological function of circRNA1002

**Figure 4:**
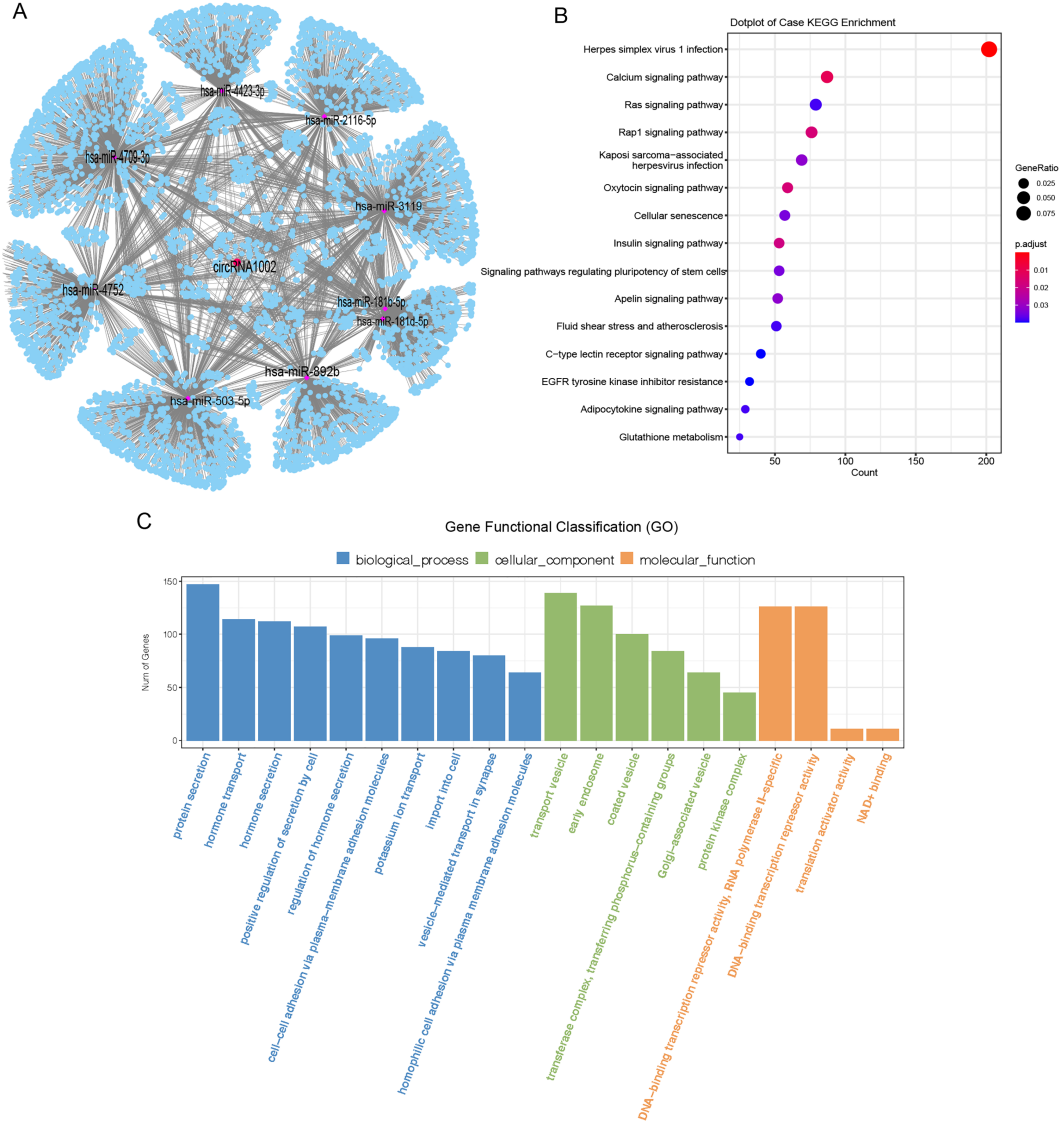
Bioinformatic analysis of circRNA1002 with sequencing data. (A) CircRNA-miRNA-mRNA network based on circRNA1002. Red dots represent circRNAs, purple dots represent miRNAs, blue dots represent mRNAs. (B) KEGG enrichment analysis of genes related to circRNA1002. (C) Go enrichment analysis of genes related to circRNA1002.

CircRNAs mainly act as molecular sponges of miRNAs to regulate targeted mRNA and affect the pathological process of diseases (Han et al., 2017). To understand the potential function of circRNA1002 in HCC, a circRNA-miRNA-mRNA network was predicted based on Targetscan and miRanda. Nine miRNAs (hsa-miR-181b-5p, hsa-miR-181d-5p, hsa-miR-2116-5p, hsa-miR-3119, hsa-miR-4423-3p, hsa-miR-4709-3p, hsa-miR-4752, hsa-miR-503-5p, and hsa-miR-892b) and various downstream mRNAs were identified to be regulated by circRNA1002 (Fig. 4A, Tables S8 and S9). A total of 5,489 circRNA1002-associated genes were identified based on Targetscan and miRDB (Fig. 4A). KEGG enrichment analysis showed that genes were enriched in various pathways, including the oxytocin signaling pathway (Fig. 4B, Table S10). Harricharran & Ogunwobi (2019) found that although the oxytocin signaling pathway is mostly known for its involvement in the female reproductive system, it also plays a role in HCC. This result indicated that circRNA1002 might affect HCC progression through genes involved in the oxytocin signaling pathway. GO enrichment analysis (Fig. 4C, Table S11) identified genes that were enriched in GO terms related to the hormone pathway and cell-cell interaction process, such as “hormone secretion” (GO:0046879), “hormone transport” (GO:0009914), and “cell-cell adhesion *via* plasma-membrane adhesion molecules” (GO:0098742). This suggested that their roles in regulating cellular adhesion and secretion are involved in HCC (Chakraborty et al., 2015).

**Figure 5:**
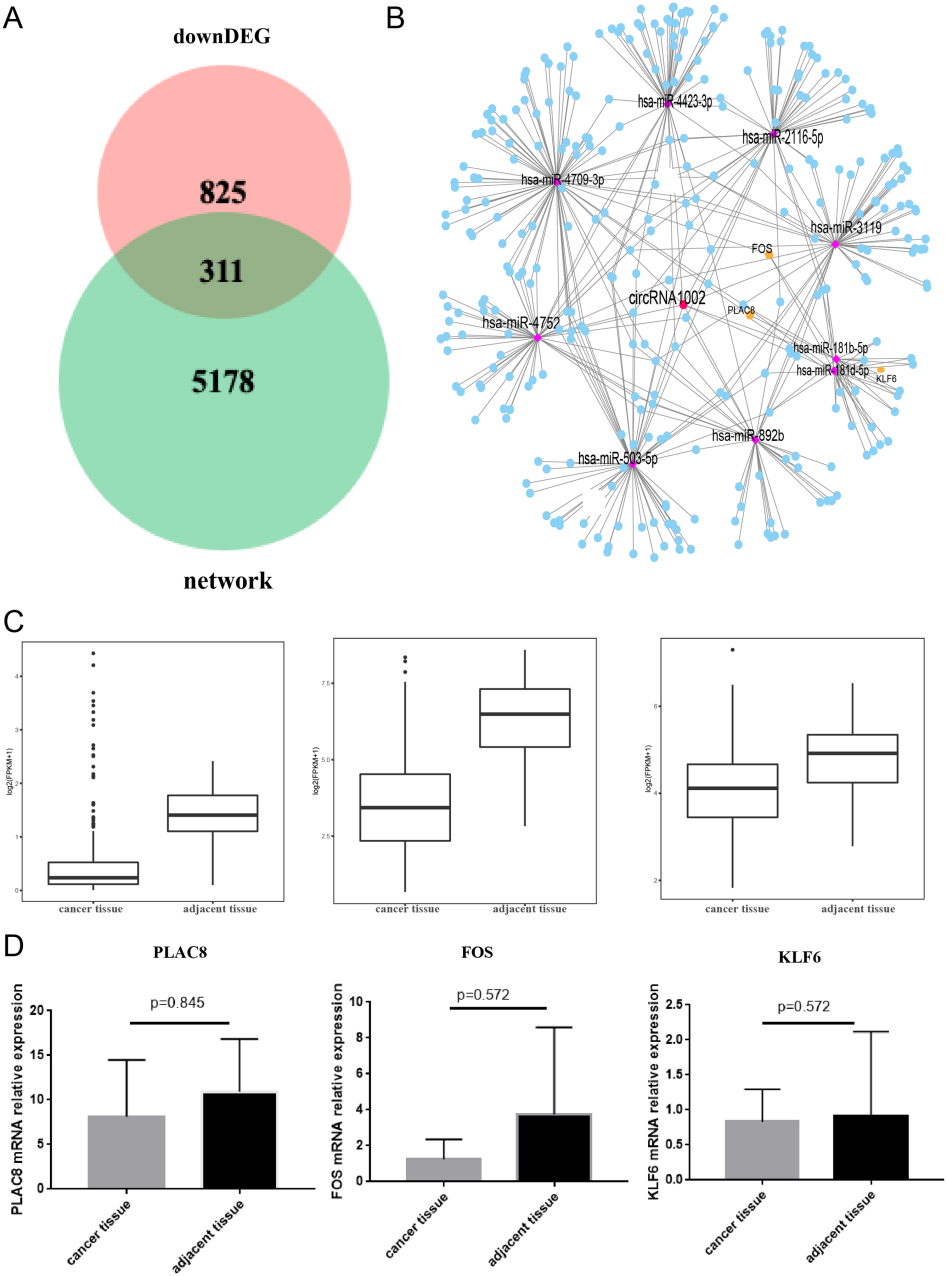
Comprehensive bioinformatic analysis of circRNA1002 with TCGA dataset. (A) Venn plot shows 311 candidate genes regulated by circRNA1002 based on sequencing data and TCGA data. (B) Subnetwork with 311 candidate genes regulated by circRNA1002 from circRNA-miRNA-mRNA network. (C) Expression level of PLAC8, FOS, and KLF6 in primary tumor tissue and normal tissue from TCGA dataset (FDR < 0.05, fold change > 2). (D) RT-qPCR validation of circRNA1002 downstream mRNAs in HCC (*n* = 6, respectively).

To further understand the biological function of circRNA1002 in HCC, we investigated the expression of genes in HCC based on the TCGA. We found that there were 1,136 down-regulated genes in HCC. To obtain the most valuable genes, we took an intersection of the previous 5,489 genes and these 1,136 genes. Ultimately, 311 genes were identified as circRNA1002-associdated genes (Fig. 5A). We further investigated the expression patterns of these genes (Fig. 5B). Based on comprehensive analysis of our previous results and literature review, we selected three HCC-related mRNAs (PLAC8, KLF6, and FOS) for further validation (Zou et al., 2016; Dang et al., 2019; Sydor et al., 2020; Sydor et al., 2020). TCGA analysis showed that these three mRNAs were statistically different, which we further evaluated using R package with a threshold of false discovery rate (FDR) < 0.05 and fold change >2 (Fig. 5C). Though RT-qPCR validation did not show statistical differences between the three genes, they presented a downregulated trend in liver cancer tissue (Fig. 5D).

## Discussion

HCC is the most common cause of death in patients with chronic liver disease, especially in China where HBV infection is common (Fu et al., 2019; Kulik & El-Serag, 2019; Sartorius et al., 2019). Many HBV cases develop into HCC. However, only a few patients are eligible for surgical intervention to obtain a better prognosis (Sun et al., 2020) as most patients receive a late diagnosis. In this study, we investigated the circRNA expression profiles of HCC patients and HBV controls in order to find a meaningful biomarker for the early detection of HCC.

CircRNAs can act as biomarkers in some diseases because of their stability, long half-life, resistance to exonucleolytic RNA decay, and evolutionary conservation (Fu et al., 2018; Qiu et al., 2019a). Many studies have confirmed the diagnostic role of circRNAs in HCC. For example, circRHOT1 was upregulated in HCC tissues, and its expression levels were associated with the clinical stage of HCC (Wang et al., 2019a). CircCAMSAP1 expression was significantly increased in HCC tissues and was involved in HCC through the miR-1294/GRAMD1A pathway (Luo et al., 2021). Tissue biopsy is the golden standard of cancer detection but it has several limitations. Biopsy is an invasive test that is accompanied by risks of bleeding and infection. Tissue biopsies only represent localized malignant changes and do not reflect the overall condition. It is inconvenient to repeat tissue biopsies in order to monitor disease progression. To solve these problems, serum circRNA biomarkers have also been studied. CircSMARCA5 is expressed at lower levels in HCC patients than in healthy controls with high discriminatory accuracy (AUC = 0.938) (Li et al., 2019b). Additionally, hsa_circ_0027089, hsa_circ_0000976, hsa_circ_0007750, and hsa_circ_0139897 were identified as potential biomarkers to distinguish HCC from healthy controls (Yu et al., 2020; Zhu et al., 2020). However, due to the heterogeneity of the samples, the expression level of circRNA may be variable. Research conducted only on selected sample types may be inadequate at identifying a more reliable serum biomarker. In this study, we analyzed circRNA expression profiles in whole blood samples of HCC and HBV patients *via* RNA-Seq. Considering that the whole blood samples included a large percentage of white blood cells, we extracted circRNAs that most likely originated from white blood cells. While these circRNAs may not have been produced by tumor cells, this had no bearing on our results based on the fact that we collected blood from both HBV-related HCC patients and HBV asymptomatic carriers, and made comparisons of the differentially expressed genes between the two groups. Our results revealed that circRNA7941, circRNA39338, and circRNA44142 were statistically different, but this relationship was not consistent across different samples. However, circRNA1002 was verified to be down-regulated in both liver tissue and blood, indicating its novelty and potential to act as a stable and convenient diagnostic marker for HCC.

The circRNAs participated in HCC through different biological processes, including proliferation, apoptosis, and metastasis (Qiu et al., 2019b). We analyzed the biological functions of circRNA1002. GO enrichment analysis revealed that “regulation of hormone secretion” and “transportation of hormone” were the most meaningful biological process terms. HCC is sexually dimorphic in both rodents and humans, and it is more common in males. This effect is dependent on sex hormones. The role of sex hormones has been verified in HCC. Li et al. (2019a) reported that androgen could act as a new target for HCC intervention through TERT. Foxa1 and foxa2 were also reported to affect the process of HCC through the estrogen or androgen receptors (Li et al., 2012). Several studies have found a relationship between circRNA and sex hormones in cancers. For example, circFNTA affects the bladder cancer process through the androgen receptor and the androgen/ADAR2/circFNTA/miR-370-3p/FNTA/KRAS axis acting as a potential pathway (Chen et al., 2020a). CircHIAT1 also plays a role in clear cell renal cell carcinoma (ccRCC), and the androgen receptor exerted its function by altering the circ HIAT1/miR-195-5p/29a/29c-3p/CDC42 pathway (Wang et al., 2017b). However, the relationships among circRNA, sex hormones, and HCC remains unknown. Our result illustrates a novel regulatory element in the HCC process.

CircRNAs may also act as miRNA sponges to regulate target gene expression. Due to the limited sample size in our study, we also conducted a comprehensive analysis combined with the TCGA database. We found circRNA1002 had the potential to harbor several miRNAs, including hsa-miR-503-3p, hsa-miR-181b-5p, hsa-miR-6715a-3p, hsa-miR-892b, and hsa-miR-181d-5p. These miRNAs are involved in the progression of various cancers. Tian et al. (2018) found that the upregulation of miR-181 may contribute to breast cancer progression through the inhibition of SPRY4 expression. Wu et al. (2017) reported that hsa-miR-181b-5p was upregulated in non-functioning invasive pituitary adenomas. Ma et al. (2020) showed that hsa-miR-892b was upregulated in pancreatic cancer. Zhang & Ge (2019) concluded that hsa-mir-6715a was related to cholangiocarcinoma. We also predicted the downstream mRNAs of circRNA1002 with further validation. Our results indicated that PLAC8 was a downstream mRNA of circRNA1002. A previous study suggested that PLAC8 affects HCC progression *via* miR-185-5p/PLAC8/β-catenin axis, providing insight for further HCC detection (Zou et al., 2016). The Fos proto-oncogene (FOS) is one of the downstream mRNAs that was repressed by ZNF382 to exert its tumor-suppressor functions (Dang et al., 2019). However, because of the small sample size and the complicated regulation of genes, RT-qPCR validation did not show a statistical significance among the genes, and we cannot definitively state that circRNA1002 plays a role in HCC *via* these genes. This study had several limitations. The sample size was small and more samples will be required to validate the findings. Also, this study was based on bioinformatic analysis and further *in vivo* and *in vitro* experiments are needed to clarify the potential molecular mechanisms between circRNA1002 and its downstream miRNAs and mRNAs. Third, considering the relatively small number of differential genes obtained in this study, some genes that play important biological functions may have been ignored. We selected genes for validation based on our previous research. Future studies need to further explore the differential genes identified in this study.

## Conclusion

This study revealed the circRNA expression profiles of HCC and HBV asymptomatic carriers. Four circRNAs, particularly circRNA1002, appeared to participate in the pathogenesis of HCC. Our study first determined that circRNA1002 may act as a stable serum biomarker in HCC since it showed a consistent trend in both blood and tissue. Additional samples are needed to corroborate the precise role of circRNA1002, but our results suggest a new method for HCC detection that is more convenient than liver tissue biopsy.

## Supplemental Information

10.7717/peerj.13640/supp-1 Supplemental Information 1 Raw data of RT-qPCR and circRNA quality validation files.

10.7717/peerj.13640/supp-2 Supplemental Information 2 Supplemental Figures and Tables.

## Additional Information and Declarations

### DNA Deposition

The following information was supplied regarding the deposition of DNA sequences: Name: CircRNA profiles in hepatitis B virus-related hepatocellular carcinoma. Project number: PRJNA751702

### Data Availability

The following information was supplied regarding data availability: The raw data are available in the Supplemental File.
