# Supplementary figures and images for: The potential of CircRNA1002 as a biomarker in hepatitis B virus-related hepatocellular carcinoma
Source: PeerJ. 2022 Jun 28;10:e13640. doi: 10.7717/peerj.13640 (PMC9248787; doi:10.7717/peerj.13640)

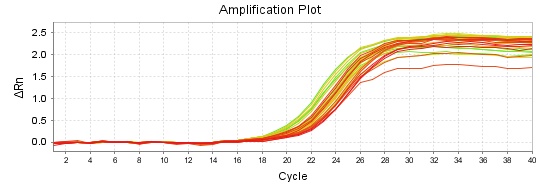

Supplement: Supplemental Information 1 [file peerj-10-13640-s001.zip › raw data/RT-qpcr/RT-qPCR circRNA/RT-qPCR circRNA serum/amplification and melting curves/Amplification Plot-GAPDH.jpg]

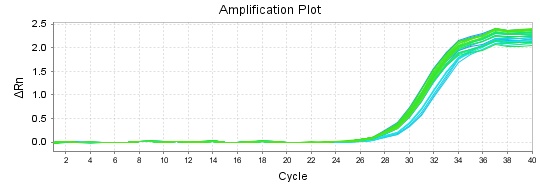

Supplement: Supplemental Information 1 [file peerj-10-13640-s001.zip › raw data/RT-qpcr/RT-qPCR circRNA/RT-qPCR circRNA serum/amplification and melting curves/Amplification Plot-circRNA1002.jpg]

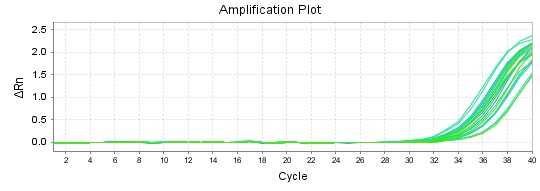

Supplement: Supplemental Information 1 [file peerj-10-13640-s001.zip › raw data/RT-qpcr/RT-qPCR circRNA/RT-qPCR circRNA serum/amplification and melting curves/Amplification Plot-circRNA26265.jpg]

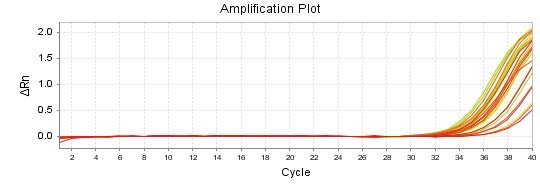

Supplement: Supplemental Information 1 [file peerj-10-13640-s001.zip › raw data/RT-qpcr/RT-qPCR circRNA/RT-qPCR circRNA serum/amplification and melting curves/Amplification Plot-circRNA26796.jpg]

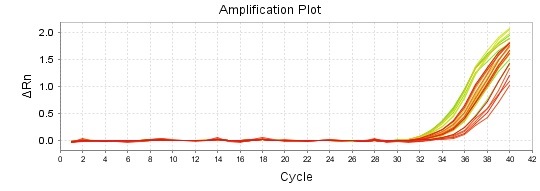

Supplement: Supplemental Information 1 [file peerj-10-13640-s001.zip › raw data/RT-qpcr/RT-qPCR circRNA/RT-qPCR circRNA serum/amplification and melting curves/Amplification Plot-circRNA36484.jpg]

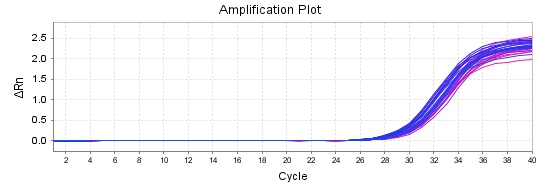

Supplement: Supplemental Information 1 [file peerj-10-13640-s001.zip › raw data/RT-qpcr/RT-qPCR circRNA/RT-qPCR circRNA serum/amplification and melting curves/Amplification Plot-circRNA39338.jpg]

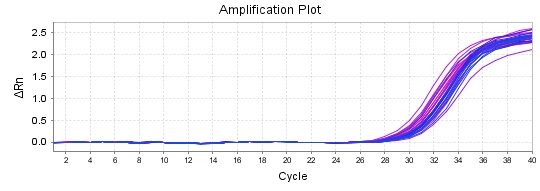

Supplement: Supplemental Information 1 [file peerj-10-13640-s001.zip › raw data/RT-qpcr/RT-qPCR circRNA/RT-qPCR circRNA serum/amplification and melting curves/Amplification Plot-circRNA44142.jpg]

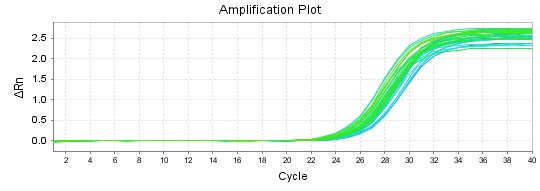

Supplement: Supplemental Information 1 [file peerj-10-13640-s001.zip › raw data/RT-qpcr/RT-qPCR circRNA/RT-qPCR circRNA serum/amplification and melting curves/Amplification Plot-circRNA4910.jpg]

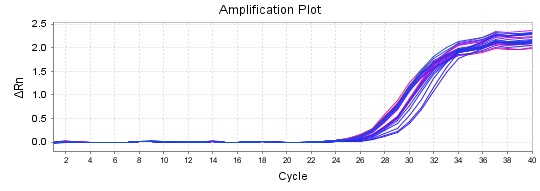

Supplement: Supplemental Information 1 [file peerj-10-13640-s001.zip › raw data/RT-qpcr/RT-qPCR circRNA/RT-qPCR circRNA serum/amplification and melting curves/Amplification Plot-circRNA7935.jpg]

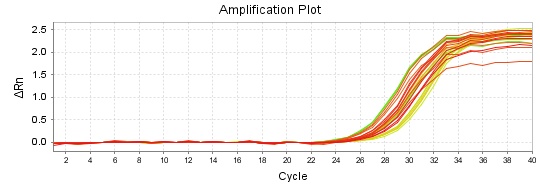

Supplement: Supplemental Information 1 [file peerj-10-13640-s001.zip › raw data/RT-qpcr/RT-qPCR circRNA/RT-qPCR circRNA serum/amplification and melting curves/Amplification Plot-circRNA7941.jpg]

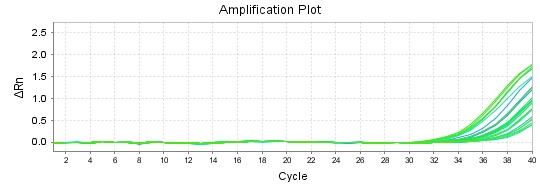

Supplement: Supplemental Information 1 [file peerj-10-13640-s001.zip › raw data/RT-qpcr/RT-qPCR circRNA/RT-qPCR circRNA serum/amplification and melting curves/Amplification Plot-hsa_circ_0007259.jpg]

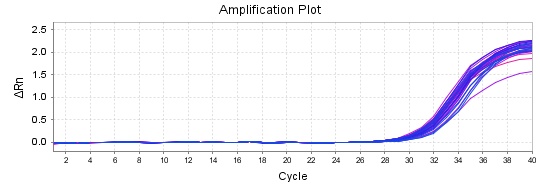

Supplement: Supplemental Information 1 [file peerj-10-13640-s001.zip › raw data/RT-qpcr/RT-qPCR circRNA/RT-qPCR circRNA serum/amplification and melting curves/Amplification Plot-hsa_circ_0072433.jpg]

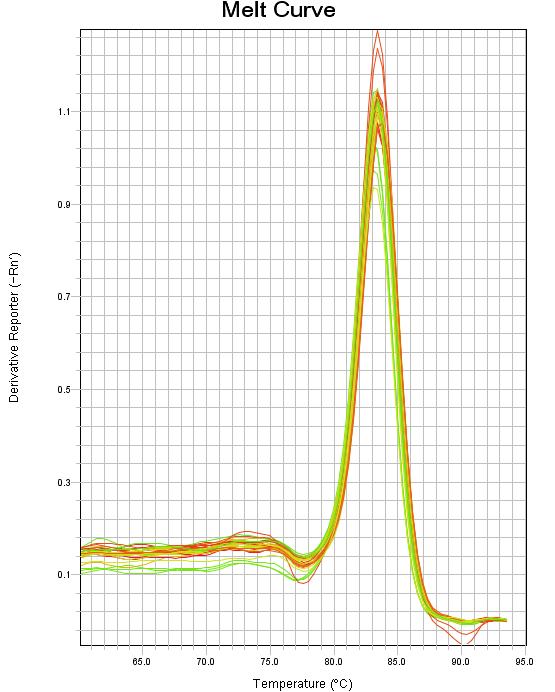

Supplement: Supplemental Information 1 [file peerj-10-13640-s001.zip › raw data/RT-qpcr/RT-qPCR circRNA/RT-qPCR circRNA serum/amplification and melting curves/Melt Curve-GAPDH.jpg]

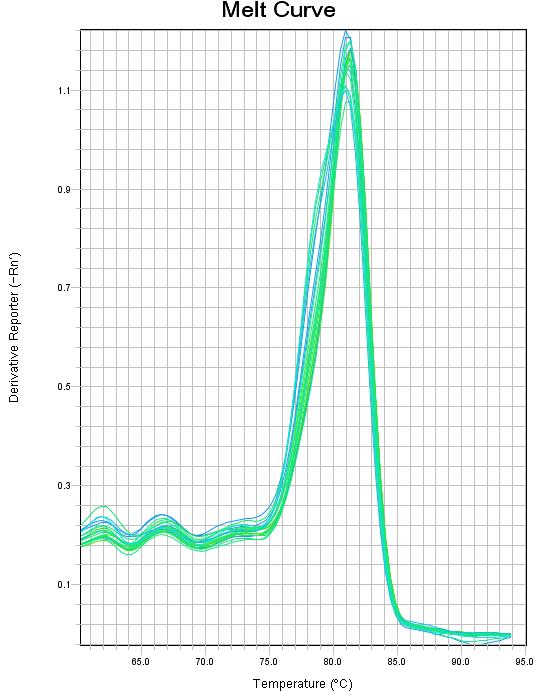

Supplement: Supplemental Information 1 [file peerj-10-13640-s001.zip › raw data/RT-qpcr/RT-qPCR circRNA/RT-qPCR circRNA serum/amplification and melting curves/Melt Curve-circRNA1002.jpg]

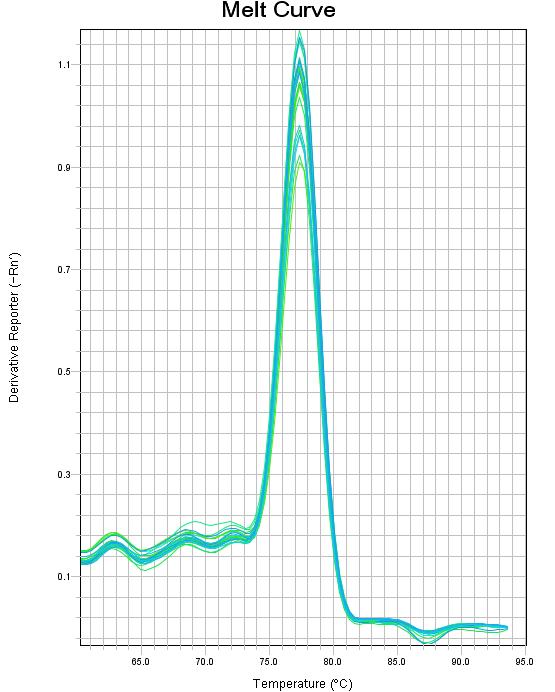

Supplement: Supplemental Information 1 [file peerj-10-13640-s001.zip › raw data/RT-qpcr/RT-qPCR circRNA/RT-qPCR circRNA serum/amplification and melting curves/Melt Curve-circRNA26265.jpg]

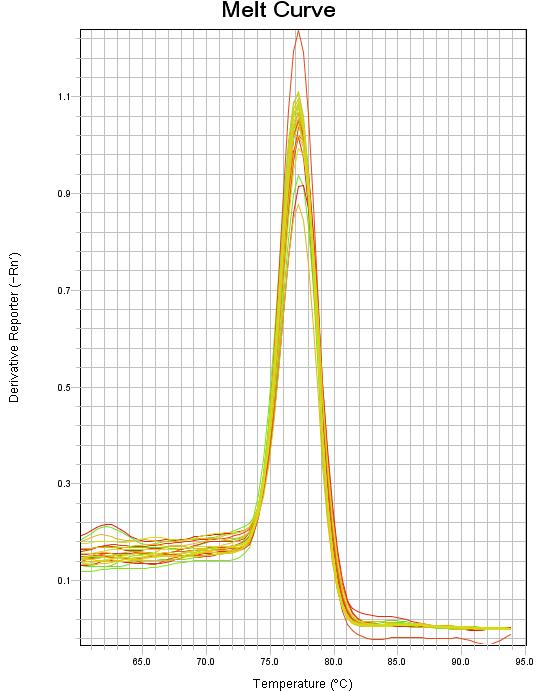

Supplement: Supplemental Information 1 [file peerj-10-13640-s001.zip › raw data/RT-qpcr/RT-qPCR circRNA/RT-qPCR circRNA serum/amplification and melting curves/Melt Curve-circRNA26796.jpg]

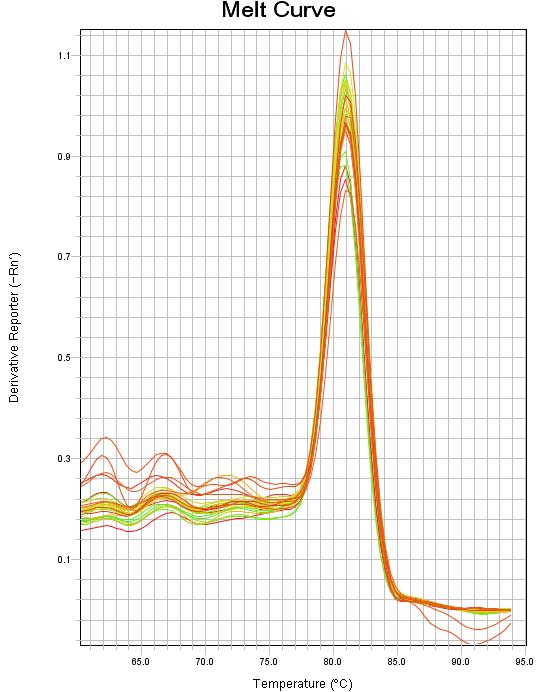

Supplement: Supplemental Information 1 [file peerj-10-13640-s001.zip › raw data/RT-qpcr/RT-qPCR circRNA/RT-qPCR circRNA serum/amplification and melting curves/Melt Curve-circRNA36484.jpg]

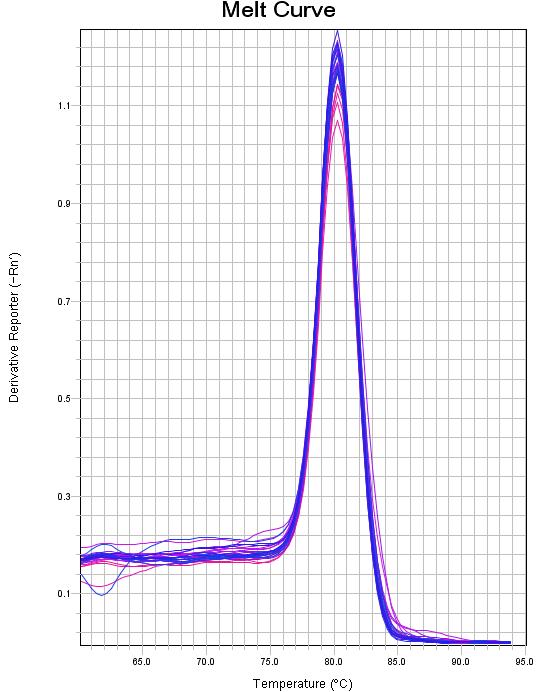

Supplement: Supplemental Information 1 [file peerj-10-13640-s001.zip › raw data/RT-qpcr/RT-qPCR circRNA/RT-qPCR circRNA serum/amplification and melting curves/Melt Curve-circRNA39338.jpg]

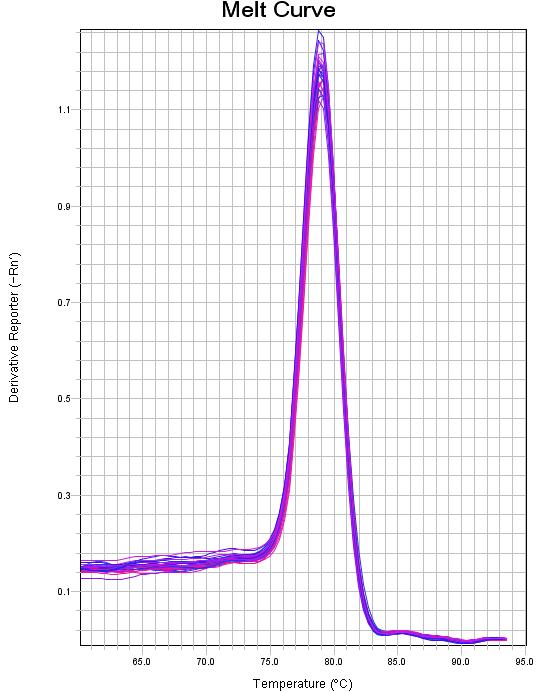

Supplement: Supplemental Information 1 [file peerj-10-13640-s001.zip › raw data/RT-qpcr/RT-qPCR circRNA/RT-qPCR circRNA serum/amplification and melting curves/Melt Curve-circRNA44142.jpg]

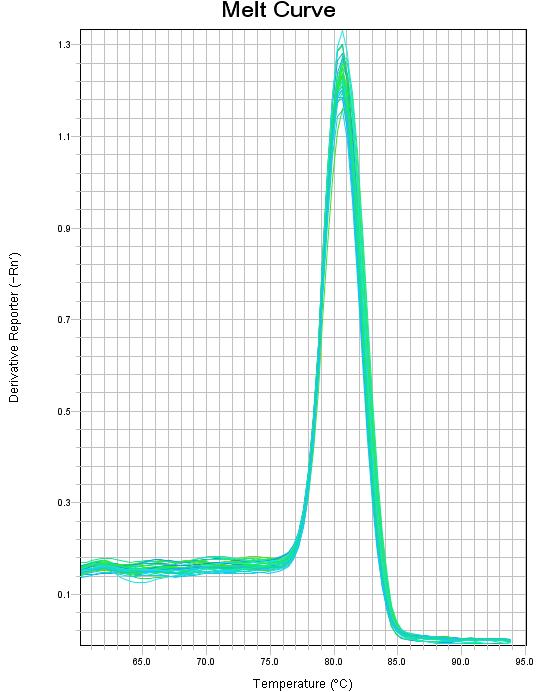

Supplement: Supplemental Information 1 [file peerj-10-13640-s001.zip › raw data/RT-qpcr/RT-qPCR circRNA/RT-qPCR circRNA serum/amplification and melting curves/Melt Curve-circRNA4910.jpg]

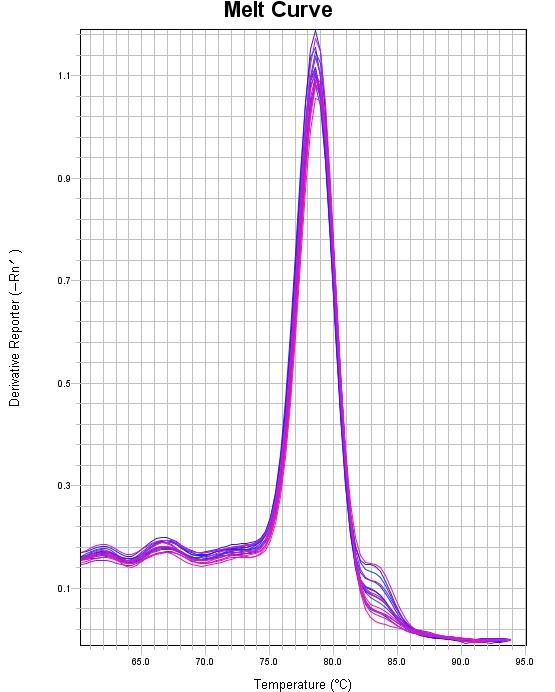

Supplement: Supplemental Information 1 [file peerj-10-13640-s001.zip › raw data/RT-qpcr/RT-qPCR circRNA/RT-qPCR circRNA serum/amplification and melting curves/Melt Curve-circRNA7935.jpg]

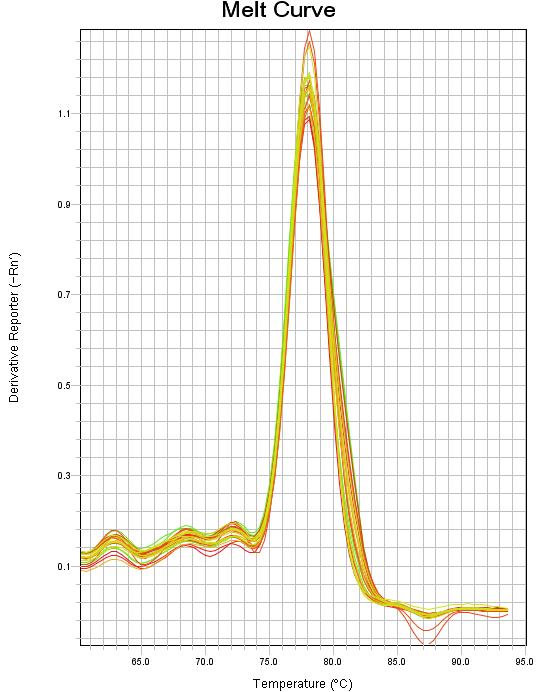

Supplement: Supplemental Information 1 [file peerj-10-13640-s001.zip › raw data/RT-qpcr/RT-qPCR circRNA/RT-qPCR circRNA serum/amplification and melting curves/Melt Curve-circRNA7941.jpg]

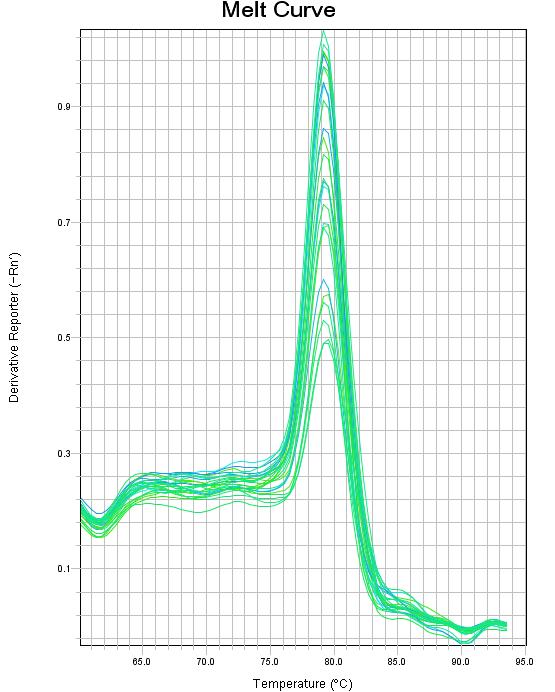

Supplement: Supplemental Information 1 [file peerj-10-13640-s001.zip › raw data/RT-qpcr/RT-qPCR circRNA/RT-qPCR circRNA serum/amplification and melting curves/Melt Curve-hsa_circ_0007259.jpg]

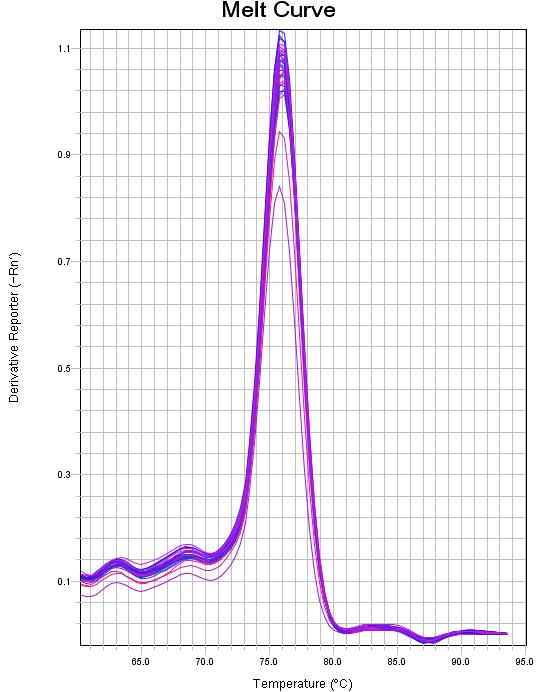

Supplement: Supplemental Information 1 [file peerj-10-13640-s001.zip › raw data/RT-qpcr/RT-qPCR circRNA/RT-qPCR circRNA serum/amplification and melting curves/Melt Curve-hsa_circ_0072433.jpg]

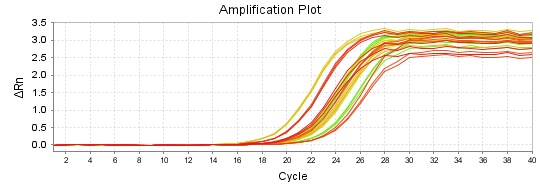

Supplement: Supplemental Information 1 [file peerj-10-13640-s001.zip › raw data/RT-qpcr/RT-qPCR circRNA/RT-qPCR circRNA tissue/amplification and melting curves/Amplification Plot-GAPDH.jpg]

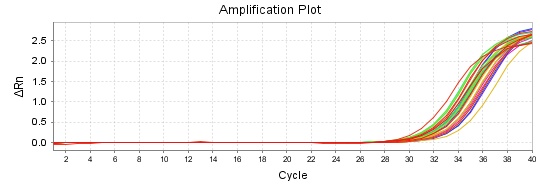

Supplement: Supplemental Information 1 [file peerj-10-13640-s001.zip › raw data/RT-qpcr/RT-qPCR circRNA/RT-qPCR circRNA tissue/amplification and melting curves/Amplification Plot-circRNA1002.jpg]

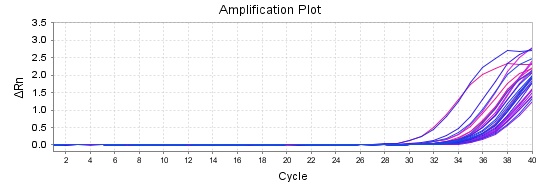

Supplement: Supplemental Information 1 [file peerj-10-13640-s001.zip › raw data/RT-qpcr/RT-qPCR circRNA/RT-qPCR circRNA tissue/amplification and melting curves/Amplification Plot-circRNA26265.jpg]

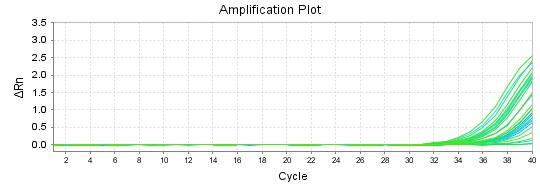

Supplement: Supplemental Information 1 [file peerj-10-13640-s001.zip › raw data/RT-qpcr/RT-qPCR circRNA/RT-qPCR circRNA tissue/amplification and melting curves/Amplification Plot-circRNA26796.jpg]

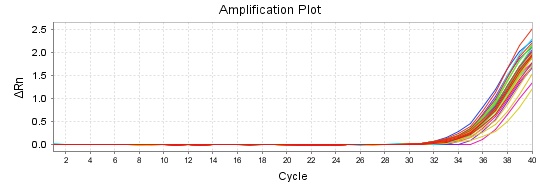

Supplement: Supplemental Information 1 [file peerj-10-13640-s001.zip › raw data/RT-qpcr/RT-qPCR circRNA/RT-qPCR circRNA tissue/amplification and melting curves/Amplification Plot-circRNA36484.jpg]

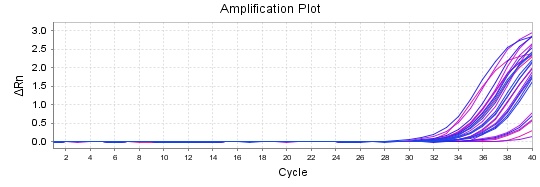

Supplement: Supplemental Information 1 [file peerj-10-13640-s001.zip › raw data/RT-qpcr/RT-qPCR circRNA/RT-qPCR circRNA tissue/amplification and melting curves/Amplification Plot-circRNA39338.jpg]

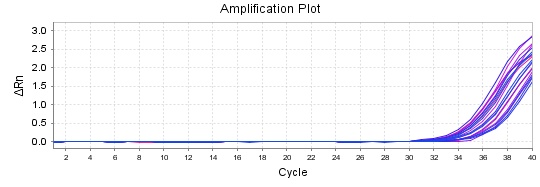

Supplement: Supplemental Information 1 [file peerj-10-13640-s001.zip › raw data/RT-qpcr/RT-qPCR circRNA/RT-qPCR circRNA tissue/amplification and melting curves/Amplification Plot-circRNA44142.jpg]

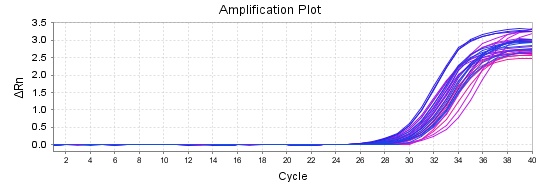

Supplement: Supplemental Information 1 [file peerj-10-13640-s001.zip › raw data/RT-qpcr/RT-qPCR circRNA/RT-qPCR circRNA tissue/amplification and melting curves/Amplification Plot-circRNA4910.jpg]

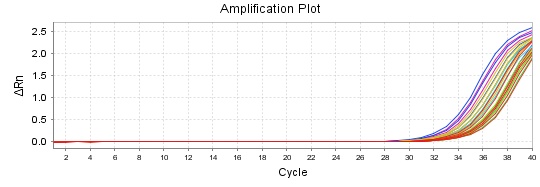

Supplement: Supplemental Information 1 [file peerj-10-13640-s001.zip › raw data/RT-qpcr/RT-qPCR circRNA/RT-qPCR circRNA tissue/amplification and melting curves/Amplification Plot-circRNA7935.jpg]

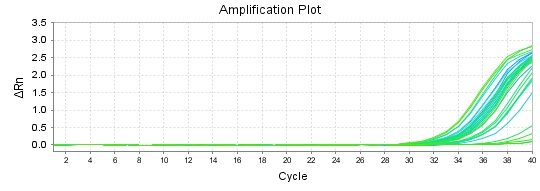

Supplement: Supplemental Information 1 [file peerj-10-13640-s001.zip › raw data/RT-qpcr/RT-qPCR circRNA/RT-qPCR circRNA tissue/amplification and melting curves/Amplification Plot-circRNA7941.jpg]

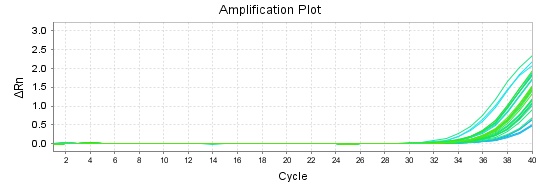

Supplement: Supplemental Information 1 [file peerj-10-13640-s001.zip › raw data/RT-qpcr/RT-qPCR circRNA/RT-qPCR circRNA tissue/amplification and melting curves/Amplification Plot-hsa_circ_0007259.jpg]

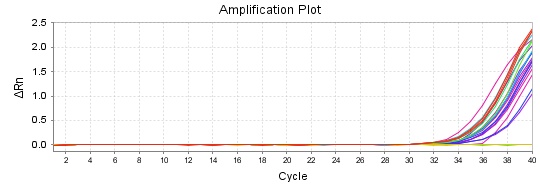

Supplement: Supplemental Information 1 [file peerj-10-13640-s001.zip › raw data/RT-qpcr/RT-qPCR circRNA/RT-qPCR circRNA tissue/amplification and melting curves/Amplification Plot-hsa_circ_0072433.jpg]

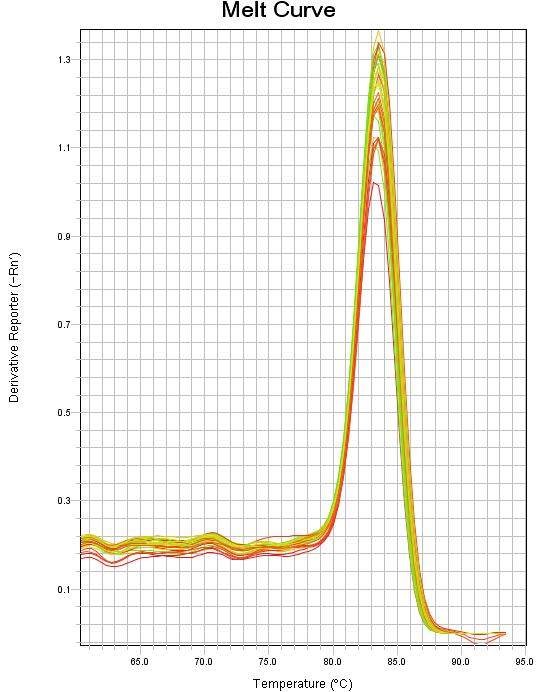

Supplement: Supplemental Information 1 [file peerj-10-13640-s001.zip › raw data/RT-qpcr/RT-qPCR circRNA/RT-qPCR circRNA tissue/amplification and melting curves/Melt Curve-GAPDH.jpg]

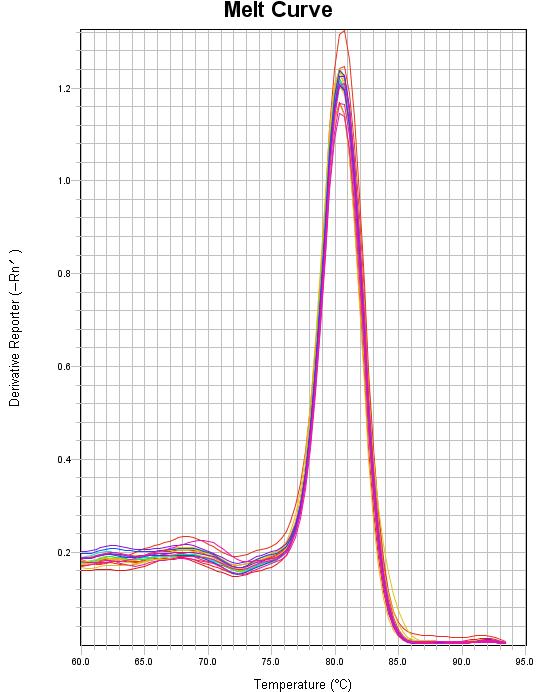

Supplement: Supplemental Information 1 [file peerj-10-13640-s001.zip › raw data/RT-qpcr/RT-qPCR circRNA/RT-qPCR circRNA tissue/amplification and melting curves/Melt Curve-circRNA1002.jpg]

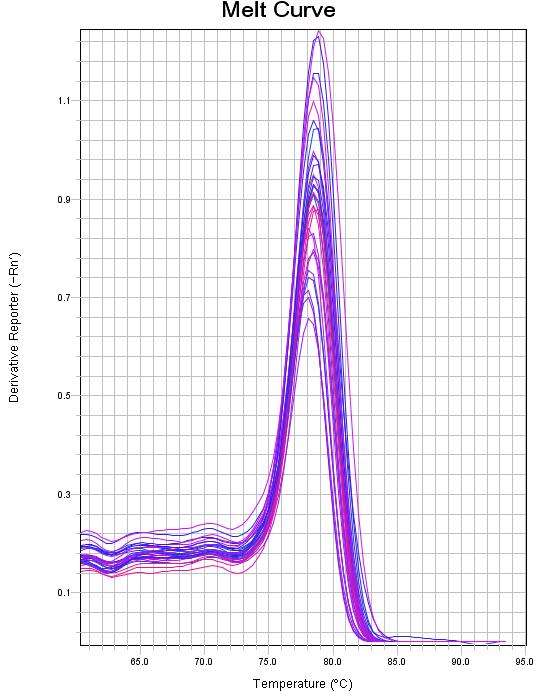

Supplement: Supplemental Information 1 [file peerj-10-13640-s001.zip › raw data/RT-qpcr/RT-qPCR circRNA/RT-qPCR circRNA tissue/amplification and melting curves/Melt Curve-circRNA26265.jpg]

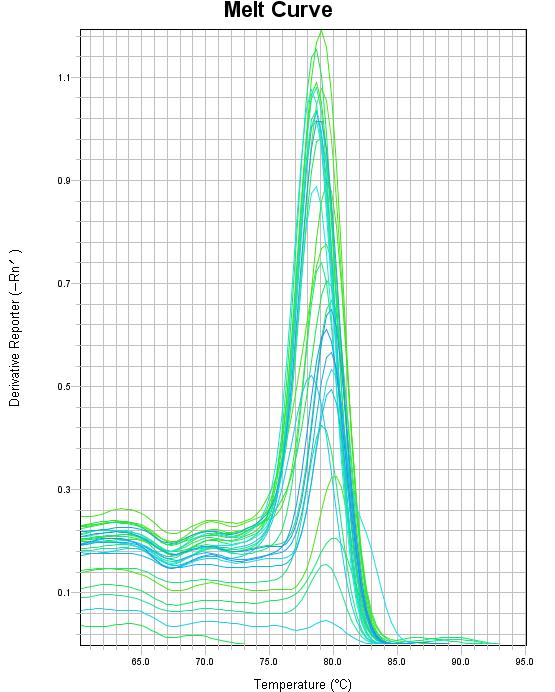

Supplement: Supplemental Information 1 [file peerj-10-13640-s001.zip › raw data/RT-qpcr/RT-qPCR circRNA/RT-qPCR circRNA tissue/amplification and melting curves/Melt Curve-circRNA26796.jpg]

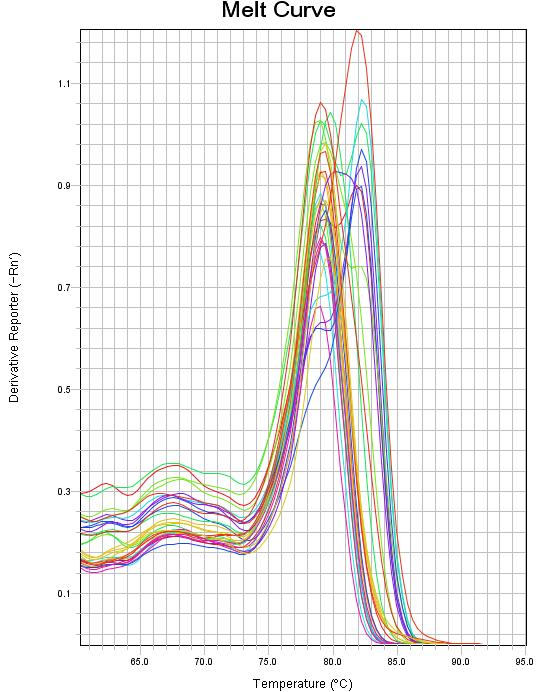

Supplement: Supplemental Information 1 [file peerj-10-13640-s001.zip › raw data/RT-qpcr/RT-qPCR circRNA/RT-qPCR circRNA tissue/amplification and melting curves/Melt Curve-circRNA36484.jpg]

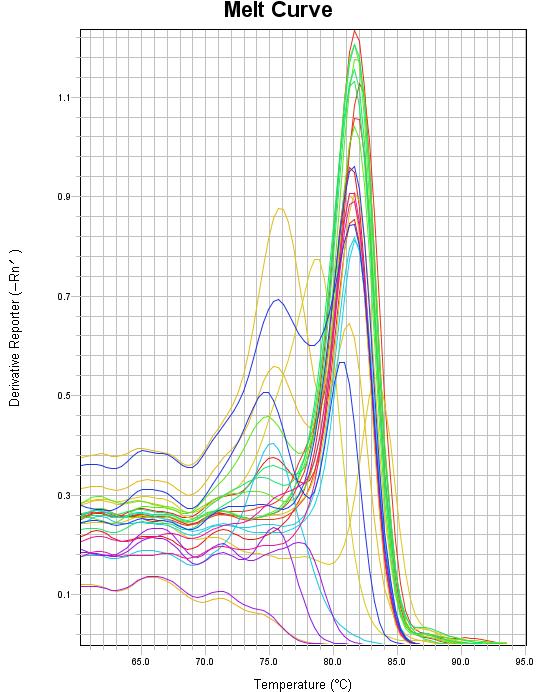

Supplement: Supplemental Information 1 [file peerj-10-13640-s001.zip › raw data/RT-qpcr/RT-qPCR circRNA/RT-qPCR circRNA tissue/amplification and melting curves/Melt Curve-circRNA39338.jpg]

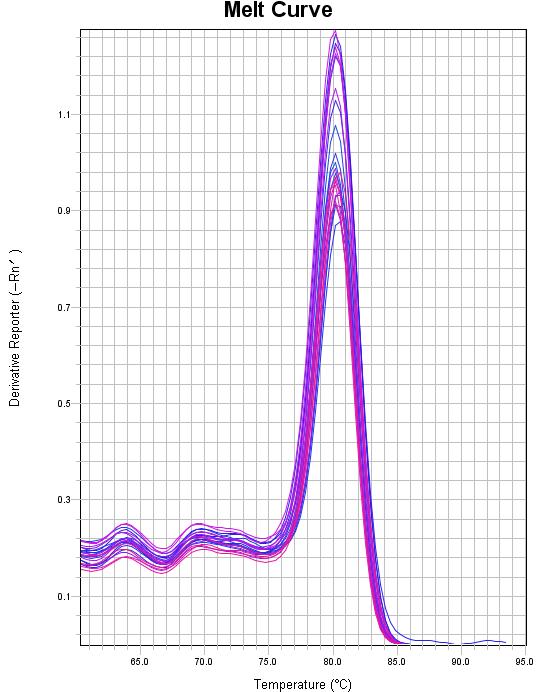

Supplement: Supplemental Information 1 [file peerj-10-13640-s001.zip › raw data/RT-qpcr/RT-qPCR circRNA/RT-qPCR circRNA tissue/amplification and melting curves/Melt Curve-circRNA44142.jpg]

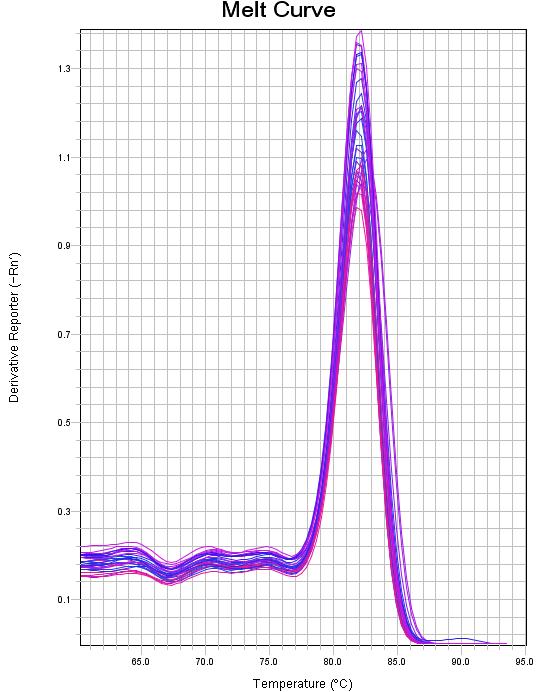

Supplement: Supplemental Information 1 [file peerj-10-13640-s001.zip › raw data/RT-qpcr/RT-qPCR circRNA/RT-qPCR circRNA tissue/amplification and melting curves/Melt Curve-circRNA4910.jpg]

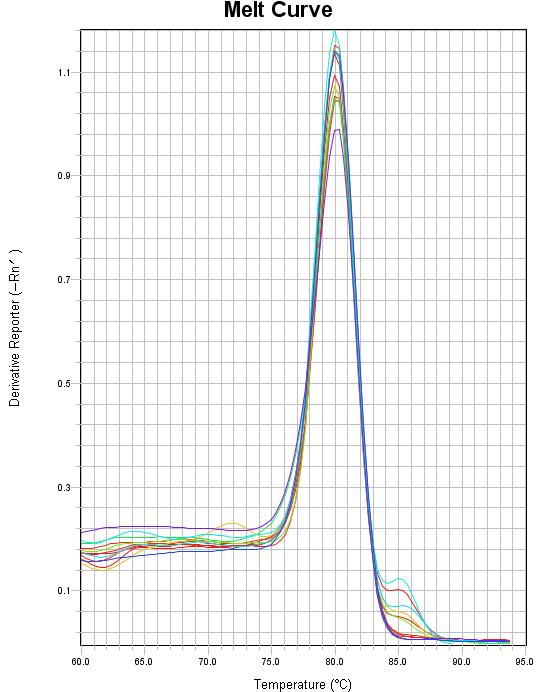

Supplement: Supplemental Information 1 [file peerj-10-13640-s001.zip › raw data/RT-qpcr/RT-qPCR circRNA/RT-qPCR circRNA tissue/amplification and melting curves/Melt Curve-circRNA7935.jpg]

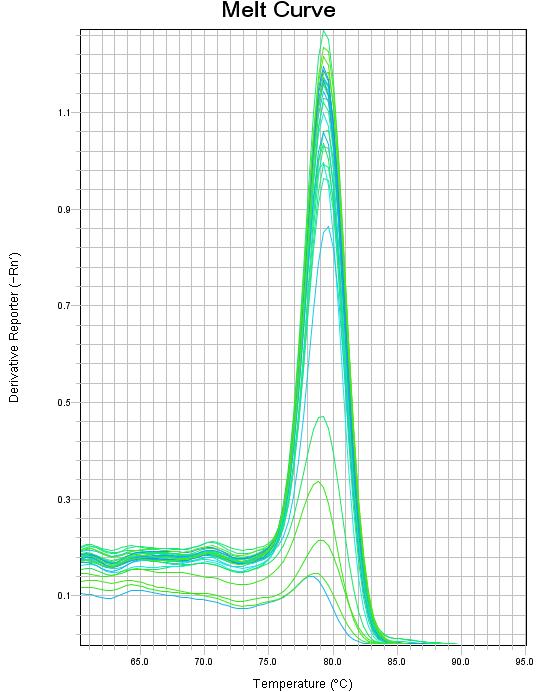

Supplement: Supplemental Information 1 [file peerj-10-13640-s001.zip › raw data/RT-qpcr/RT-qPCR circRNA/RT-qPCR circRNA tissue/amplification and melting curves/Melt Curve-circRNA7941.jpg]

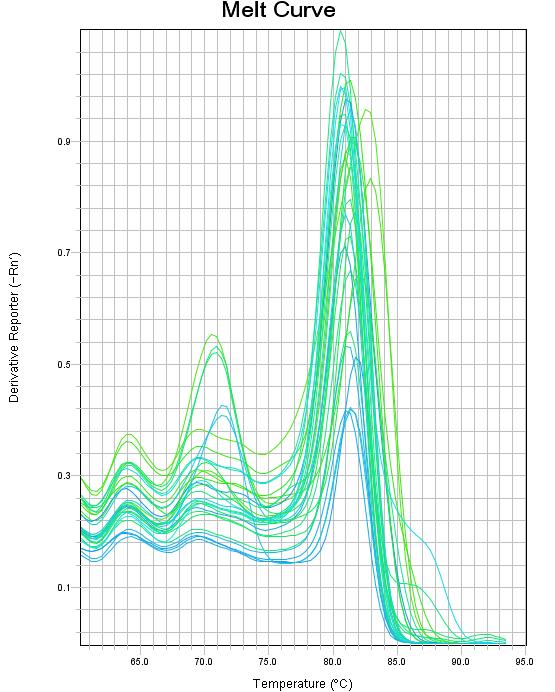

Supplement: Supplemental Information 1 [file peerj-10-13640-s001.zip › raw data/RT-qpcr/RT-qPCR circRNA/RT-qPCR circRNA tissue/amplification and melting curves/Melt Curve-hsa_circ_0007259.jpg]

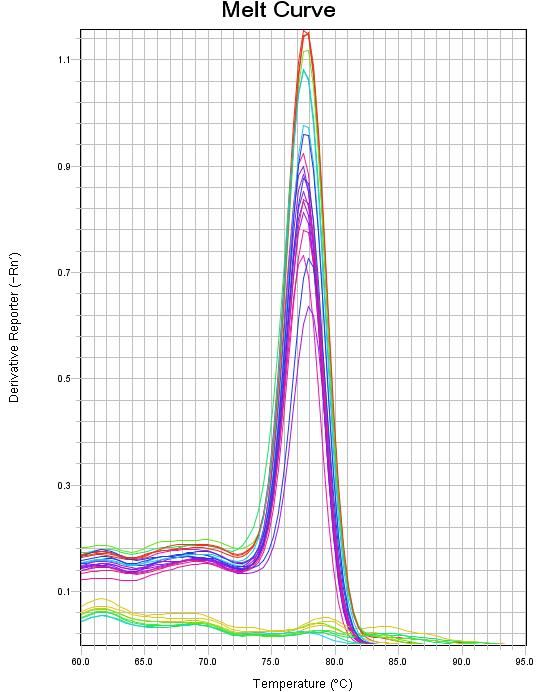

Supplement: Supplemental Information 1 [file peerj-10-13640-s001.zip › raw data/RT-qpcr/RT-qPCR circRNA/RT-qPCR circRNA tissue/amplification and melting curves/Melt Curve-hsa_circ_0072433.jpg]

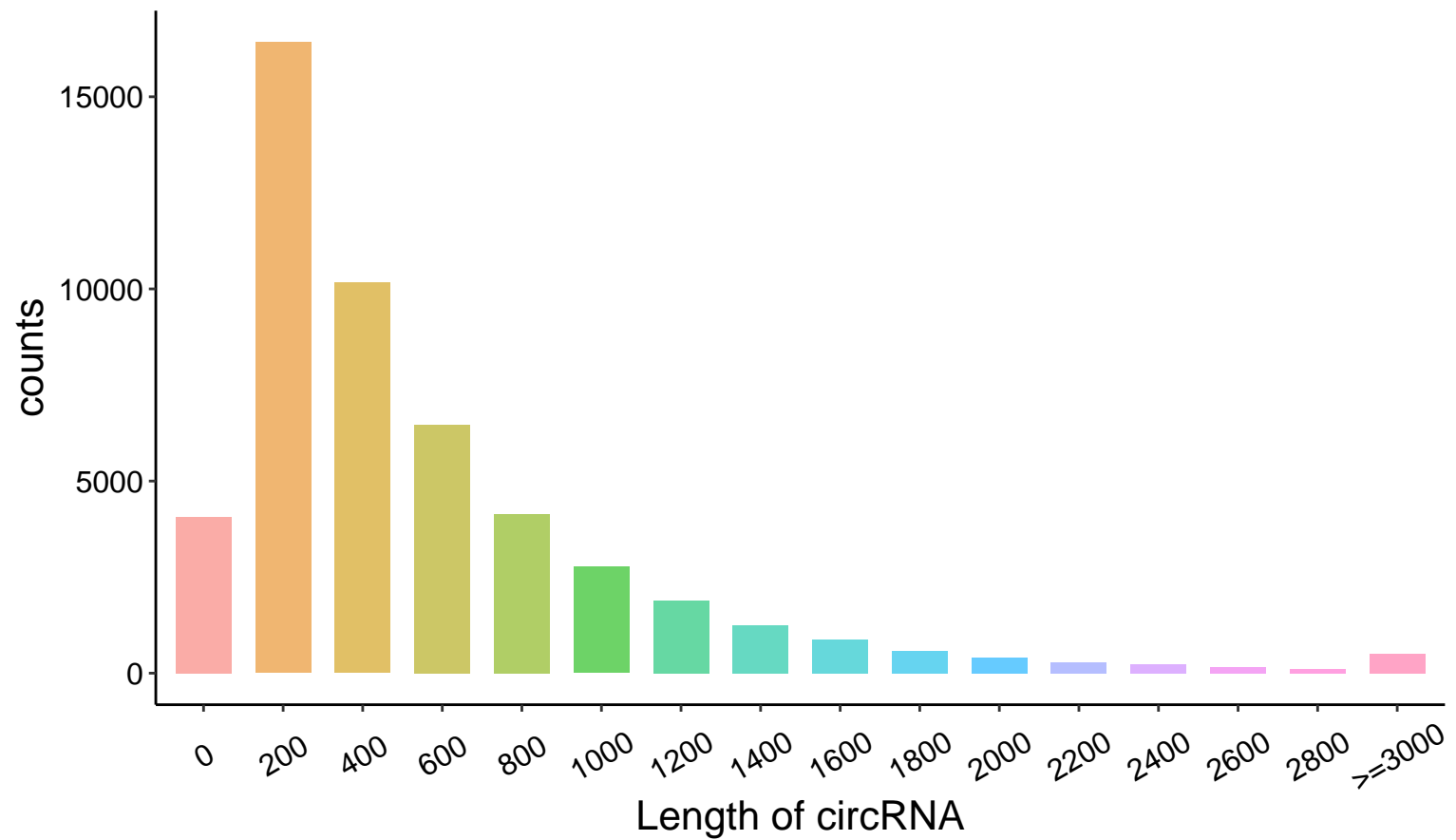

Supplement: Supplemental Information 1 [file peerj-10-13640-s001.zip › raw data/circRNA quality validation files/circRNA length.pdf]

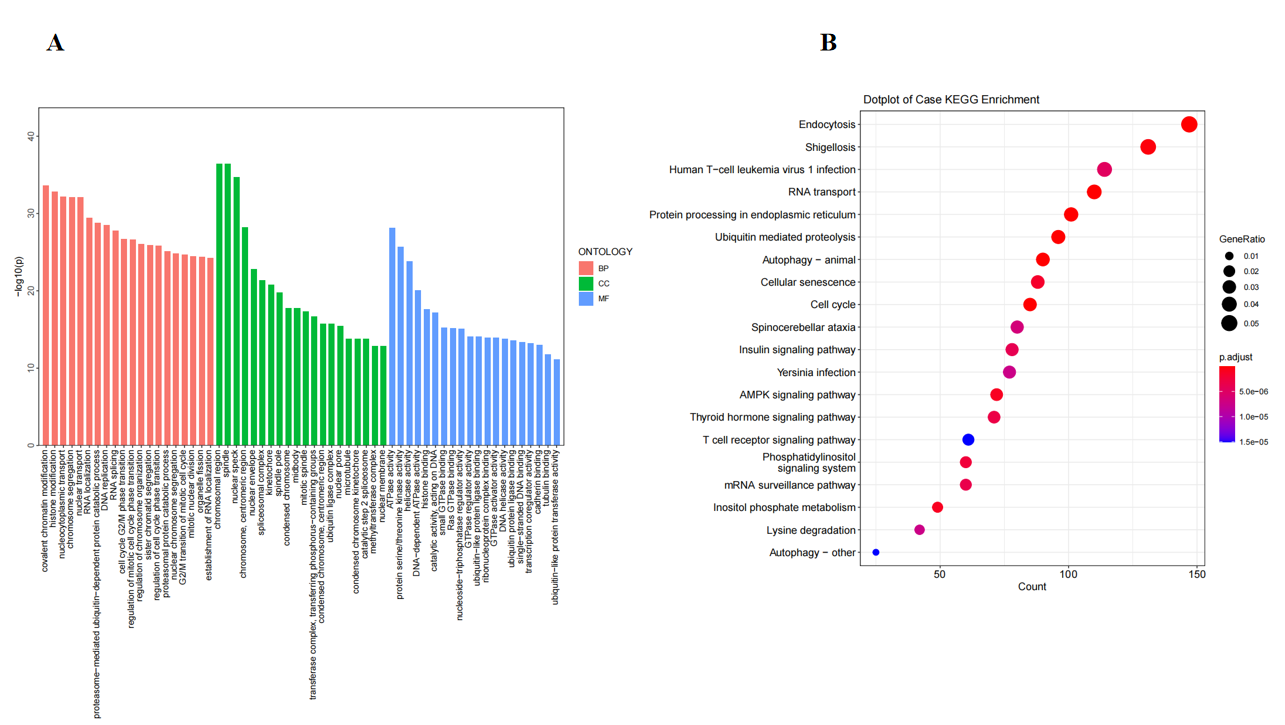

Supplement: Supplemental Information 2 [file peerj-10-13640-s002.zip › Supplementary data/FigS1.PNG]
