# Supplementary material for: The potential of CircRNA1002 as a biomarker in hepatitis B virus-related hepatocellular carcinoma
Source: PeerJ. 2022 Jun 28;10:e13640. doi: 10.7717/peerj.13640 (PMC9248787; doi:10.7717/peerj.13640)
Supplement: Supplemental Information 2 [file peerj-10-13640-s002.zip › Supplementary data/TableS1.doc]

Table.S1 Detailed information of 30 HCC patients and HBV asymptomatic carriers

| Cases | Samples | Gend；er | Age | Tumor number | TNM stage | HBV-DNA | Tumor size* | ALT (u/L) | AST (u/L) | AFP  (ng/ml) |
| --- | --- | --- | --- | --- | --- | --- | --- | --- | --- | --- |
| HCC |  |  |  |  |  |  |  |  |  |  |
| 1  2  3  4  5 | blood | male | 42 | 1 | I | 10^7 | 2 | 68.3 | 110.1 | <0.24 |
|  | blood | male | 55 | 2 | II | 10^6 | 4 | 248.5 | 305.3 | 3.18 |
|  | blood | male | 45 | 1 | I | 2*10^5 | 1 | 32.7 | 65.9 | 262.8 |
|  | blood | female | 45 | 2 | II | 80 | 3 | 21.1 | 27.6 | 10890 |
|  | blood | male | 46 | 2 | II | 10^5 | 3 | 30.7 | 27.1 | 683.37 |
| 6  7  8  9  10  11  12  13  14  15  16  17  18  19  20  21  HBV  1  2  3  4 | blood | male | 41 | 2 | II | 10^6 | 3 | 30.1 | 27.1 | <0.25 |
|  | blood | female | 58 | 1 | I | 80 | 1 | 39.2 | 43.2 | 790.3 |
|  | blood | female | 48 | 1 | I | ＜10 | 2 | 20.1 | 12.1 | 102 |
|  | blood | male | 60 | 1 | I | 10^3 | 4 | 46.2 | 50.2 | 3.50 |
|  | blood | male | 55 | 1 | I | 200 | 1 | 38.1 | 30.1 | 80.1 |
|  | tissue | female | 39 | 1 | I | 70 | 2 | 63.1 | 60.2 | 900.2 |
|  | tissue | female | 58 | 2 | III | 50 | 6 | 30.1 | 23.3 | 35.2 |
|  | tissue | female | 40 | 1 | I | 10^5 | 1.8 | 55.1 | 54.3 | 100.2 |
|  | tissue | male | 57 | 2 | II | 10^5 | 3.5 | 35.1 | 32.1 | 702.1 |
|  | tissue | female | 42 | 2 | III | 10^5 | 6 | 50.1 | 43.1 | 450.2 |
|  | tissue | male | 61 | 2 | II | 10^6 | 3 | 180.1 | 200.2 | 120.3 |
|  | tissue | female | 51 | 1 | I | ＜10 | 2 | 100.2 | 120 | 592.1 |
|  | tissue | male | 49 | 1 | I | 2*10^5 | 2 | 92.7 | 89.2 | 200.6 |
|  | tissue | female | 43 | 2 | II | 180 | 1.5 | 52.2 | 54.1 | 310.2 |
|  | tissue | female | 45 | 2 | III | 30 | 6 | 13.1 | 11.1 | 201.1 |
|  | tissue | female | 54 | 2 | II | 250 | 3 | 38.2 | 33.1 | 211.2 |
|  |  |  |  |  |  |  |  |  |  |  |
|  | blood | male | 40 | - | - | 10^6 | - | 24.1 | 25.0 | <0.25 |
|  | blood | female | 47 | - | - | 2*10^5 | - | 89 | 78 | 3.23 |
|  | blood | female | 40 | - | - | 20 | - | 21.7 | 13.8 | 2.51 |
|  | blood | female | 45 | - | - | 17 | - | 13.9 | 14.5 | 2.01 |
| 5  6  7  8  9 | blood  blood  blood  blood  blood | male  male  male  male  male | 46  47  49  43  53 | -  -  -  -  - | -  -  -  -  - | 10^6  100  ＜10  10^5  60 | -  -  -  -  - | 80  64.3  30  51  14.8 | 76.1  66.1  38  58  21.4 | 2.50  9.21  3.05  3.43  5.25 |

* If the number of tumors is greater than 1, it represents the larger tumor.
