# Supplementary material for: The potential of CircRNA1002 as a biomarker in hepatitis B virus-related hepatocellular carcinoma
Source: PeerJ. 2022 Jun 28;10:e13640. doi: 10.7717/peerj.13640 (PMC9248787; doi:10.7717/peerj.13640)
Supplement: Supplemental Information 2 [file peerj-10-13640-s002.zip › Supplementary data/TableS6.doc]

**TableS6. Sanger sequencing results**

**circRNA7941**

***backsplice junction***

**circRNA26265**

***backsplice junction***

**hsa_circ_0072433**

***backsplice junction***

**circRNA26796**

***backsplice junction***

**circRNA4910**

***backsplice junction***

**circRNA39338**

***backsplice junction***

**hsa_circ_0007259**

***backsplice junction***

**circRNA44142**

***backsplice junction***

**circRNA36484**

***backsplice junction***

**circRNA1002**

***backsplice junction***

**circRNA7935**

***backsplice junction***
