# Supplementary material for: The potential of CircRNA1002 as a biomarker in hepatitis B virus-related hepatocellular carcinoma
Source: PeerJ. 2022 Jun 28;10:e13640. doi: 10.7717/peerj.13640 (PMC9248787; doi:10.7717/peerj.13640)
Supplement: Supplemental Information 2 [file peerj-10-13640-s002.zip › Supplementary data/TableS7.doc]

**Primers used in RT-qPCR validation experiments**

|  | Forward primer | Reverse primer |
| --- | --- | --- |
| GAPDH | GAPDH-F  GCTGAGAACGGGAAGCTTGT | GAPDH-R  GACTCCACGACGTACTCAGC |
| circRNA7941 | circRNA7941-F  GCTACCCTTTTGGGCCTTTA | circRNA7941-R  CACTACAAAGTTGACTTGTG |
| circRNA26265 | circRNA26265-F  CTACAGGACCTGAAGTCTTT | circRNA26265-R  GGGCAGTTATGTCATCATCT |
| circRNA35761 (hsa_circ_0072433) | hsa_circ_0072433-F  GGCCCTATGCTTAGGATAAT | hsa_circ_0072433-R  TGGATAAGCAACAGTTAATG |
| circRNA26796 | circRNA26796-F  GTCTAGTTTAAAGGCTGTTC | circRNA26796-R  CCTCTGGTATGCAGATAATG |
| circRNA4910 | circRNA4910-F  GCCAGAAGATGCACACTTGA | circRNA4910-R  TCCAAACACTTCCGTCTGTC |
| circRNA39338 | circRNA39338-F  AACTTTTCAGTGGCAAGCTG | circRNA39338-R  AGCCCCCAACTTGGAGAAAT |
| circRNA364 (hsa_circ_0007259) | hsa_circ_0007259-F  CAAAGTGTTGGAGGACAGAC | hsa_circ_0007259-R  AAGTAAATGACACCTTTTTA |
| circRNA44142 | circRNA44142-F  AGCCAATCCCCAGATGTTCC | circRNA44142-R  CTTCCACTTTAGGTCACTTA |
| circRNA36484 | circRNA36484-F  AAGACCCTGGAGAAGATGTG | circRNA36484-R  AAATGATATCCCTGCTCAAT |
| circRNA1002 | circRNA1002-F  CAGTCAACAAAAGTTGTGGA | circRNA1002-R  AGGCACCTTTCTAACAGATT |
| circRNA7935  PLAC8  FOS    KLF6 | circRNA7935-F  TCTCATCACCATGGGGAAAA  PLAC8-F  GTGTGACTGTTTCAGCGACTG  FOS-F  CCGGGGATAGCCTCTCTTACT  KLF6-F  GGCAACAGACCTGCCTAGAG | circRNA7935-R  AGGATGGATGCTTGCTACTC  PLAC8-R  CTGCAACTTGACACCCAAGG  FOS-R  CCAGGTCCGTGCAGAAGTC  KLF6-R  CTCCCGAGCCAGAATGATTTT |
